# Local and regional dynamics of chikungunya virus transmission in Colombia: the role of mismatched spatial heterogeneity

**DOI:** 10.1186/s12916-018-1127-2

**Published:** 2018-08-30

**Authors:** Sean M. Moore, Quirine A. ten Bosch, Amir S. Siraj, K. James Soda, Guido España, Alfonso Campo, Sara Gómez, Daniela Salas, Benoit Raybaud, Edward Wenger, Philip Welkhoff, T. Alex Perkins

**Affiliations:** 1 Department of Biological Sciences and Eck Institute for Global Health, University of Notre Dame, Notre Dame, IN USA; 2 Mathematical Modelling of Infectious Diseases Unit, Institut Pasteur, 75015 Paris, France; 3 CNRS UMR2000: Génomique évolutive, modélisation et santé (GEMS), Institut Pasteur, Paris, France; 4 Center of Bioinformatics, Biostatistics and Integrative Biology, Institut Pasteur, 75015 Paris, France; 5 Subdirección de Análisis de Riesgo y Respuesta Inmediata en Salud Pública, Instituto Nacional de Salud de Colombia, Bogotá, Colombia; 6 Grupo de Enfermedades Transmisibles, Instituto Nacional de Salud de Colombia, Bogotá, Colombia; 7 Institute for Disease Modeling, Bellevue, WA USA

**Keywords:** *Aedes aegypti*, Aggregation bias, Arbovirus, Chikungunya, Colombia, Epidemic, Mathematical model, Spatial scale, Transmission dynamics

## Abstract

**Background:**

Mathematical models of transmission dynamics are routinely fitted to epidemiological time series, which must inevitably be aggregated at some spatial scale. Weekly case reports of chikungunya have been made available nationally for numerous countries in the Western Hemisphere since late 2013, and numerous models have made use of this data set for forecasting and inferential purposes. Motivated by an abundance of literature suggesting that the transmission of this mosquito-borne pathogen is localized at scales much finer than nationally, we fitted models at three different spatial scales to weekly case reports from Colombia to explore limitations of analyses of nationally aggregated time series data.

**Methods:**

We adapted the recently developed Disease Transmission Kernel (DTK)-Dengue model for modeling chikungunya virus (CHIKV) transmission, given the numerous similarities of these viruses vectored by a common mosquito vector. We fitted versions of this model specified at different spatial scales to weekly case reports aggregated at different spatial scales: (1) single-patch national model fitted to national data; (2) single-patch departmental models fitted to departmental data; and (3) multi-patch departmental models fitted to departmental data, where the multiple patches refer to municipalities within a department. We compared the consistency of simulations from fitted models with empirical data.

**Results:**

We found that model consistency with epidemic dynamics improved with increasing spatial granularity of the model. Specifically, the sum of single-patch departmental model fits better captured national-level temporal patterns than did a single-patch national model. Likewise, multi-patch departmental model fits better captured department-level temporal patterns than did single-patch departmental model fits. Furthermore, inferences about municipal-level incidence based on multi-patch departmental models fitted to department-level data were positively correlated with municipal-level data that were withheld from model fitting.

**Conclusions:**

Our model performed better when posed at finer spatial scales, due to better matching between human populations with locally relevant risk. Confronting spatially aggregated models with spatially aggregated data imposes a serious structural constraint on model behavior by averaging over epidemiologically meaningful spatial variation in drivers of transmission, impairing the ability of models to reproduce empirical patterns.

**Electronic supplementary material:**

The online version of this article (10.1186/s12916-018-1127-2) contains supplementary material, which is available to authorized users.

## Background

Viral diseases transmitted by mosquitoes, including dengue, Zika, chikungunya, and yellow fever, are a rapidly growing problem and together pose a risk to approximately half the world’s population [1–3]. In the past 5 years, both the Zika (ZIKV) and chikungunya (CHIKV) viruses were introduced into the Western Hemisphere and rapidly spread among naïve populations in South America, Central America, and the Caribbean, resulting in millions of cases and causing a public health crisis [4–9]. In addition, hundreds of millions of people are infected by dengue virus (DENV) each year [1]. Due to the influence of environmental conditions on DENV transmission, as well as complex immunological interactions among the four DENV serotypes, many regions experience periodic dengue epidemics [10, 11]. Faced with these large epidemics, limited resources need to be targeted towards areas with the highest transmission and the most vulnerable populations. In addition, public health officials would like to be able to predict where epidemics of these diseases may spread next [12].

Mathematical models can play a critical role in identifying at-risk populations and forecasting the course of an epidemic based on current epidemiological conditions [13–16]. Models are often fitted to time series of confirmed or suspected cases to estimate epidemiological parameters such as the reproduction number of the pathogen, which can be used to predict how rapidly the epidemic will spread or whether it is expected to die out [17–19]. For simplicity, these models often make assumptions about transmission dynamics that do not reflect biological reality [20]. One important assumption that is often made is that the human population is well mixed, which for a mosquito-transmitted pathogen means that each person within a given area has an equal chance of being bitten by any of the mosquitoes within that area [20]. The spatial scale at which this assumption is reasonable is determined primarily by the scales of both human and mosquito movement [21]. Empirical studies have shown that chikungunya clusters at scales of neighborhoods or villages [22, 23], implying that models posed at larger scales may be incompatible with the biology of CHIKV transmission.

Over large spatial scales, e.g., at the national or provincial scale, human populations are unevenly distributed, and population mixing and movement depend on transportation networks, with movement among localities affected by a number of different economic, cultural, geographical, and environmental factors [24–27]. Contact rates between humans and mosquitoes also vary considerably among locations due to the influence of meteorological variables, such as temperature, rainfall, and relative humidity, on mosquito population dynamics [28–30]. As a result of these different factors, exposure within a particular geographic region can be highly heterogeneous, with important implications for disease dynamics. For example, estimates of transmission rates made from models assuming homogeneous mixing can lead to underestimates of the level of effort needed to control the spread of a pathogen [31]. Spatial heterogeneity in human-mosquito contact rates can be incorporated into disease transmission models by subdividing the population and modeling movement between subpopulations [32]. Heterogeneity in human-mosquito contact rates between different subpopulations can be represented by explicitly modeling mosquito population dynamics based on local climate [33].

In late 2013, CHIKV was introduced into the Caribbean and soon spread throughout North and South America, infecting millions of people [13, 34]. The first confirmed cases in Colombia were reported in June 2014, and almost 500,000 cases were reported by the end of 2015. Suspected chikungunya cases were reported at the second administrative level (municipality) in Colombia throughout the epidemic, enabling examination of its spatiotemporal dynamics. By simulating the chikungunya epidemic in Colombia at different spatial scales, we examine how model assumptions about the scale of human-mosquito interactions affect the accuracy of model predictions. Specifically, we simulate disease dynamics at a finer spatial scale than the observed time series used to fit the model and compare these model results to simulations conducted at the coarser spatial scale at which surveillance data were aggregated. A comparison of model fits at different levels of spatial aggregation is used to assess how incorporating spatial heterogeneity in environmental and demographic conditions improves model accuracy and provides additional insights into the epidemiological parameters estimated during the model-fitting process. In addition, simulation results at spatial scales below the level of observation provide estimates of unobserved spatial heterogeneity in epidemic dynamics.

## Methods

### Model description

We modeled CHIKV transmission dynamics using a new extension of the Institute for Disease Modeling’s (IDM) Epidemiological Modeling Disease Transmission Kernel (EMOD-DTK) software [35]. EMOD is an individual-based disease modeling platform that supports multiple disease transmission routes, including vector-based transmission initially designed to simulate malaria transmission dynamics [35]. We modified the generic vector-transmission model to represent the transmission dynamics of arboviruses transmitted by *Aedes aegypti* mosquitoes. Modifications to the generic vector model included incorporating life-history parameters specific to *Ae. aegypti*, including parameters that capture the sensitivity of its life cycle to rainfall and temperature [36]. The modified model also includes the ability to simulate the transmission of multiple serotypes of the same pathogen; however, for CHIKV we assume that there is a single strain. Mosquito life-history parameters, as well as parameters determining the temperature-dependent frequency of feeding on humans, are described elsewhere [36].

Several parameters affecting the transmissibility of CHIKV were estimated from recent studies (Table 1). The probability of an infected individual developing a symptomatic infection was estimated as 0.72 based on the mean of estimates from 13 different studies (Table 2) [37–49]. An individual’s infectiousness, *ζ*(*t*), over the duration of infection was assumed to vary according to

1 $$ \zeta (t)={e}^{-a/{c}_3}, $$

where *a* = *c*_1_(*D*_*t*_ − *c*_2_)^2^ and *D*_*t*_ is the number of days since infection. The values for parameters *c*_1_, *c*_2_, and *c*_3_ were estimated by fitting Eq. (1) to viremia data from [50] and assuming that the dose-response curve for CHIKV was the same as a DENV curve calculated elsewhere [51]. Because another study [50] did not find any significant differences in viremias between asymptomatic and symptomatic infections, we used the same parameter values for asymptomatic and symptomatic individuals. The extrinsic incubation rate, *δ*_*T*_, for CHIKV in *Ae. aegypti* following an infected blood meal depends on the temperature (*T*) in Kelvins and was assumed to follow the Arrhenius equation, ${\delta}_T={a}_1{e}^{-{a}_2T}$, with parameters fit to the exponential representation in [52]. CHIKV-specific parameters *a*_1_ and *a*_2_ were estimated by fitting to data from [53]. We assumed that only 8% of symptomatic infections are reported, consistent with estimates for dengue [54] and similar to the 9% observed for chikungunya in Puerto Rico [38]. The total number of infections reported is the product of the symptomatic rate and the reporting rate for symptomatic infections. To ensure that our model results were not overly dependent on particular values for either the symptomatic rate or reporting rate, we conducted a sensitivity analysis by fitting the single-patch and multi-patch departmental models for six different departments with combined symptomatic and reporting rates that were 25% lower or higher than the values used in the main analysis (corresponding to a symptomatic rate of 0.54–0.9 when the reporting rate is 0.08 or a reporting rate of 0.06–0.10 when the symptomatic rate is 0.72).

**Table 1:** Estimates for key parameters affecting the transmissibility of chikungunya virus and the probability that an infection is reported. Sources are studies from which values were taken or studies that contained data that were used to estimate parameter values (see Methods for details)

Parameter	Value	Source(s)
Symptomatic probability	0.72	See Table 2
Incubation period	3 days	
Reporting probability	0.08	[54]
Infectiousness parameters		[50]
*c*_1_	0.547	
*c*_2_	3.256	
*c*_3_	1.489	
Extrinsic incubation rate		[53]
*a*_1_	9.47 × 10^12^	
*a*_2_	9550	
Transmission probability	0.5

**Table 2:** Estimates of the probability of an infected individual developing a symptomatic infection from 13 different epidemiological studies

Location	Value	Sample Size	Source
Saint Martin	0.61	42	[37]
Puerto Rico	0.625	56	[38]
Emilia-Romagna region, Italy	0.82	33	[39]
La Réunion	0.968	128	[40]
Cebu City, Philippines	0.179	106	[41]
Kerala, India	0.962	260	[42]
Lamu Island, Kenya	0.55	215	[43]
Comoros	0.857	209	[44]
Mayotte	0.723	440	[45]
La Réunion	0.833	967	[46]
Dakshina Kannada district, India	0.937	224	[47]
Bagan Panchor, Malaysia	0.825	40	[48]
Phatthalung province, Thailand	0.529	314	[49]

EMOD-DTK is capable of simulating pathogen transmission among humans and mosquitoes in a single patch, as well as spatial dynamics across multiple patches connected by human and mosquito movement. The spatial scales considered in this study are much larger than the typical dispersal distance of *Ae. aegypti* [55], so all spatial models only allowed for human movement among patches. Within a single patch, humans and mosquitoes are evenly mixed (although heterogeneous biting patterns can be implemented in the model). Mosquito population dynamics were represented by a compartmental model rather than modeled individually to reduce the computational requirements of each simulation. The compartmental model incorporates each life-history stage and simulates adult female mosquito biting and ovipositing behaviors.

CHIKV transmission was simulated in populations at three different spatial scales. First, simulations of the chikungunya epidemic for all of Colombia were run with a single patch representing the entire country. Second, single-patch simulations were run for each of the 32 departments (plus the capital district of Bogotá) individually. Finally, multi-patch simulations were run for each department (except for Bogotá, which consists of a single municipality) with separate patches for each municipality (second administrative unit in Colombia). Within a patch, various aspects of the mosquito population and the extrinsic incubation period of the virus within the mosquito are affected by local climate variables. Parameter values used in all simulations are described in Table 1. Gridded daily temperature, precipitation, and relative humidity from 2013 to 2016 were initially modeled at a 5 km × 5 km resolution [56]. The mean climate values at the country, department, and municipality scales were calculated by taking population-weighted averages of the daily values from the gridded data sets.

Due to computational constraints, the size of the human population in some simulations was either scaled down or subsampled. For the single-patch simulations at the national and departmental scales, the mosquito and human populations were both scaled to one tenth of their actual size. The populations in the multi-patch departmental model were not scaled, because the human population sizes are already smaller at the municipality level. In addition, humans were simulated using an adaptive sampling scheme, with a maximum patch population of 50,000 individuals in single-patch simulations and 20,000 in multi-patch simulations. For patches in the multi-patch simulations with fewer than 20,000 residents, everyone in the population is simulated individually. For patches with more than 20,000 residents, the patch population size is set at 20,000 humans and each individual in the simulation is weighted so as to approximate the actual population size (e.g., if the actual population size is 200,000, then each individual in the simulation receives a weighting of 10.0). To test the sensitivity of simulation results to the maximum population size used in the adaptive sampling scheme, we ran simulations for a population of 4.85 million with the maximum population size ranging from 5000 to 50,000 (the sampling factor ranged from ~ 1000:1 to 100:1). Between-simulation variance increased for maximum population sizes < 20,000, but it was not significantly reduced by increasing the maximum size above 20,000 (Additional file 1: Figure S1A). There also did not appear to be any bias in the mean incidence estimates for maximum population sizes of ≥ 20,000 (Additional file 1: Figure S1B).

### Epidemiological data and model fitting

We obtained a time series of weekly suspected cases for each municipality in Colombia from the start of the epidemic through the end of the third week of 2016 from the national system of surveillance for public health of Colombia (SIVIGILA). A suspected case was defined as a person having an acute onset of fever (> 38 °C) and severe arthralgia or arthritis not explained by other medical conditions and being a resident or having visited epidemic or endemic areas within 2 weeks prior to the onset of clinical symptoms. In the 2014–2015 period, a laboratory-confirmed case was defined as a suspected case with positive reverse transcription polymerase chain reaction (RT-PCR), and in 2016 confirmed cases included RT-PCR or positive serology.

These time series were used to estimate several model parameters separately at each spatial scale. For both the spatial and non-spatial models, we fitted the model to time series data to estimate (1) the amount of rainfall-associated temporary mosquito larval habitat in each department (2) the decay rate of this temporary habitat, and (3–5) the timing, magnitude, and duration of virus importation into the country or department. For the spatial model, we also fitted a scaling factor that modulated movement rates among municipalities. Therefore, the multi-patch departmental models involved fitting only a single additional parameter relative to the single-patch departmental models and the single-patch national model (six vs. five).

Rainfall-associated temporary larval mosquito habitat in the model increases with rainfall and decays at a rate proportional to the evaporation rate driven by temperature and humidity [35]. The amount of larval habitat is the primary driver of the number of adult mosquitoes per human in simulations. Fitting the larval habitat parameters in the model to the time series of suspected cases allowed us to estimate the ratio of adult mosquitoes per human that recreate the observed transmission dynamics. The amount of temporary rainfall habitat was scaled by the department population size, so that we could compare the relative amounts of larval habitat per person in different departments. For the multi-patch models, a single larval habitat size parameter was fitted for each department, with the amount of habitat in each municipality scaled by the municipality population size so that the amount of larval habitat per person was constant for all municipalities in the department.

The initial introduction of CHIKV was assumed to occur via a single pulse of importation with variable timing, size, and duration. We represented this pulse with a Gaussian probability density function, with the timing of the introduction represented by the mean and the duration represented by the standard deviation. We then multiplied this curve by a scaling factor representing the overall magnitude of the importation pulse [36]. The mean timing was allowed to range between the beginning of 2014 and the end of the study period (the first case in Colombia was reported in June 2014). The standard deviation was between 1 and 50 days, and the magnitude corresponded to between 0.001 to 100 expected cumulative infections, with the actual number of imported infections drawn from a Poisson distribution with a mean equal to the scaled magnitude of the Gaussian. For the spatial models, the initial imported case(s) were assumed to occur in the largest municipality in the department, with introduction into the other municipalities (patches) occurring via simulated human movement.

Movement rates among municipalities within a department were estimated using a gravity-like model [57] fitted to department-level migration rates from the most recent census, which were then downscaled to the municipality level based on population, distance, and economic covariates. These migration rates were then scaled to a short-term movement rate with an initial scaling factor that was previously estimated in a study [58] comparing census immigration rates and cellphone-based movement patterns in Kenya. Because that study was conducted in a different country and the scaling factor was very different for different travel lengths (e.g., 2.15 for daily travel but 101.92 for weekly travel), we fitted this range between 1.02 and 101.92, setting the upper bound at the high weekly movement rate seen in Kenya. These movement rates were represented in the model as the fraction of individuals in patch *i* who travel on a given day to patch *j*. Movement events are assumed to last for 1 day, with a 100% probability that the individual will return to their home patch.

Fitting of the transmission model was conducted by maximum likelihood using a gradient ascent iterative optimization algorithm called OptimTool that has been built into the EMOD-DTK software framework. The initial parameter values were drawn from the hypersphere of the specified parameter ranges, centered around an initial best guess for that parameter value with a mean search radius determined by the number of parameters and the standard deviation of the radius set at 1/10 of the mean. One hundred draws from this parameter space were conducted for each iteration of the model-fitting process. Due to the stochasticity involved in individual-based models, each sample was simulated separately four times, for a total of 400 simulations per iteration. At the end of each iteration step, the log likelihood of each sample was calculated. The number of suspected cases was assumed to be binomially distributed given the population, and, in order to incorporate uncertainty in the infection and reporting rates, the probability of a reported case was assumed to come from a beta distribution, resulting in a beta-binomial likelihood function. Initially, the beta distribution was assumed to be uninformative (*α* = 1, *β* = 1), but after simulation results became available, the beta hyperparameters were adjusted to reflect this new information via a Bayesian update. As a result, *α* = 1 + *X*_*i*_ and *β* = 1 + *N*_*i*_ - *X*_*i*_, where *N*_i_ is the population size in patch *i* and *X*_*i*_ is the average number of reported cases across simulations [59]. This process was repeated ten times, with parameter draws from each successive iteration based on the log likelihoods from all previous iterations.

The accuracies of model estimates were assessed by calculating the mean absolute scaled error (MASE) of the estimated vs. observed weekly suspected case numbers [60]. The MASE calculates the estimation error at each time step (numerator) relative to the prediction from a simple stationary autoregressive lag-1 (AR-1) model:

2 $$ MASE=\frac{1}{T}\sum \limits_{t=1}^T\frac{\left|{y}_t-{x}_t\right|}{\frac{1}{T-1}{\sum}_{t=2}^T\left|{y}_t-{y}_{t-1}\right|}, $$

where *y*_*t*_ and *x*_*t*_ are the observed and estimated numbers of cases for weeks *t* = 1,…,*T*. The relative accuracies of the single-patch vs. multi-patch models for each department were then measured by calculating the relative MASE = MASE_m_/MASE_s_.

Because the municipality-level observations were not used in the fitting process at the department level, we were able to compare these observations to the predicted municipality-level dynamics from the multi-patch models to assess the model’s capability to reproduce disease dynamics at spatial scales below the scale at which the fitting process occurred. The total number of observed cases and cumulative per capita incidence were calculated for each municipality in a department and compared to the estimated case totals and per capita incidence per municipality. Comparisons were made by calculating the Pearson’s correlation coefficient for the reported and estimated municipality values within each department using the model results from 100 best-fitting simulations per department. These municipality-level correlations were compared to correlations calculated for a null model that allocates the estimated cases in a department to each municipality within the department using a multinomial distribution with probabilities weighted by municipality population size.

## Results

### Fit to national time series

**Fig. 1:**
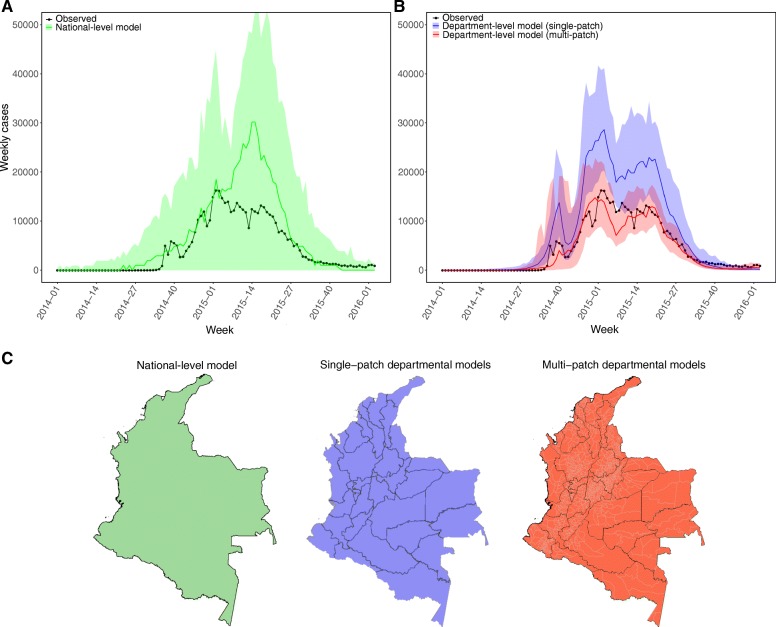
**a** Weekly number of reported chikungunya cases in Colombia (*black*), along with the mean and 95% CI from the (*green*) national-level model. **b** National-level totals derived by combining the results of each departmental model with either a (*blue*) single-patch model per department, or (*red*) the multi-patch models. **c** Maps of Colombia showing the spatial scale of the different models, with the color coding for the different models used in all figures

Between the start of 2014 and the third week of 2016, our best-fit national-level model projects a median of 873,318 (95% confidence interval (CI) 0–1,000,353) reported cases, an overestimate of the 481,284 actually reported (Fig. 1a). The 95% CI includes zero because about 19% of the time the importations did not result in any locally acquired cases. Excluding these stochastic fadeouts, the median estimate of reported cases is 886,947 (95% CI 805,164–1,010,590). The best-fit national-level model estimates matched the observations well early in the epidemic through the end of 2014 but overestimated cases following the peak in the second week of 2015, projecting a continued increase in cases until week 15 in 2015. The best-fit estimate for date of introduction was week 7 of 2014 (95% CI week 52, 2013 to week 25, 2014).

The combined total of reported cases predicted by the 33 different single-patch department-level models was 864,296 (95% CI 709,075–892,697), overestimating the observed national total by 79.6% (95% CI 47.3–85.5%). The timing of the epidemic was relatively accurate, but the size of the peak was significantly overestimated, with estimated cases during the peak week being 72.3% (95% CI 23.2–151.1%) above the observed national number of cases (Fig. 1b).

The combined total of reported cases at the national level predicted by the multi-patch department-level models was more accurate than either the national-level model or the combined total from the single-patch department-level models (Fig. 1b). The median estimate of reported cases was 451,920 (95% CI 375,139–511,009), an underestimate of 6.1% (95% CI –6.2 to 22.1%). The number of cases during the week of peak reported cases was underestimated by 11.5% (95% CI –37.0 to 45.1%), and the estimated peak was 2 weeks earlier than the observed peak. However, the estimated peak was only 9.0% below the observed peak (95% CI –40.6 to 49.6%).

### Department-level fits

**Fig. 2:**
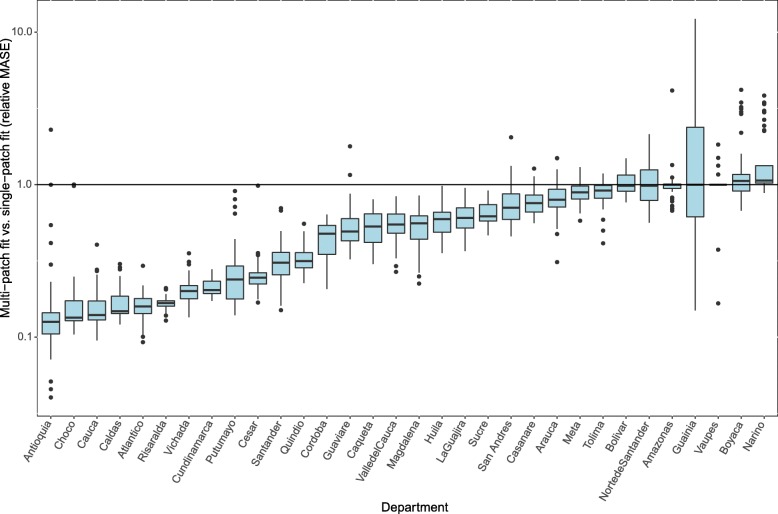
Fit of multi-patch simulations vs. single-patch simulations to department-level time series for each department in Colombia (excluding Bogotá). Relative model fit is measured via the relative mean scaled error (relMASE) of the single-patch fit to the multi-patch fit, with relMASE < 1 indicating a better fit for the multi-patch model

**Fig. 3:**
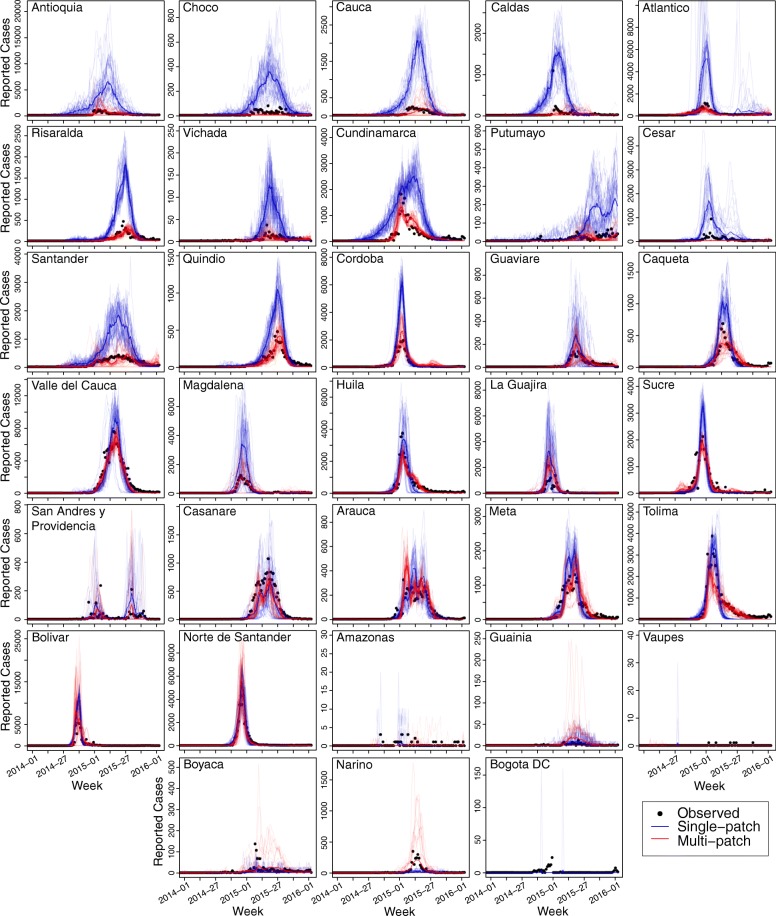
Comparisons of department-level results for single-patch and multi-patch models. *Black dots* represent the observed time series, while *blue lines* represent the 40 best-fitting individual simulations from the single-patch model and *red lines* represent the best-fitting simulations from the multi-patch model. *Darker colored blue* and *red lines* are the single best-fitting simulations

The median MASE across single-patch departmental models was 3.37 (95% CI 0.50–27.46), while the median MASE across all multi-patch departmental models was 1.75 (95% CI 0.50–6.11), for an overall relative MASE of 0.55 (95% CI 0.12–1.90). The MASE of the multi-patch model was lower than the MASE of the single-patch model for the majority of departments (Fig. 2). The 95% CI of the MASE from the single-patch model was not entirely below the MASE from the multi-patch model for any department, while it was entirely above the multi-patch model MASE for 15 departments: Atlantico (10.22–15.83 vs. 1.55–2.81), Caldas (6.7–7.76 vs. 0.95–1.92), Caqueta (3.20–4.99 vs. 1.40–2.86), Cauca (25.09–28.83 vs. 2.67–8.13), Cesar (4.41–9.06 vs. 1.57–1.87), Cordoba (4.35–6.44 vs. 1.01–3.27), Cundinamarca (5.51–6.33 vs. 1.08–1.52), Huila (1.71–3.39 vs. 1.14–1.60), Magdalena (5.72–8.74 vs. 1.64–4.92), Putumayo (3.07–12.32 vs. 1.59–2.76), Quindio (5.14–6.68 vs. 1.49–2.82), Risaralda (10.36–12.75 vs. 1.68–2.14), Santander (11.456–17.01 vs. 2.40–10.97), Valle del Cauca (1.87–4.71 vs. 1.24–1.76), and Vichada (5.26–7.86 vs. 1.06–1.96). In a few departments, the single-patch model overestimated the number of cases by a large margin while the multi-patch model provided a good fit to the observed time series (e.g., Cauca, Santander, and Risaralda; Fig. 3). In the department where the relative MASE for the multi-patch model was the poorest (Narino), the best-fit simulation from the multi-patch model actually reproduced the epidemic well, but overestimated the epidemic size in some simulations, while the single-patch model underestimated the epidemic size.

### Parameter estimates

**Fig. 4:**
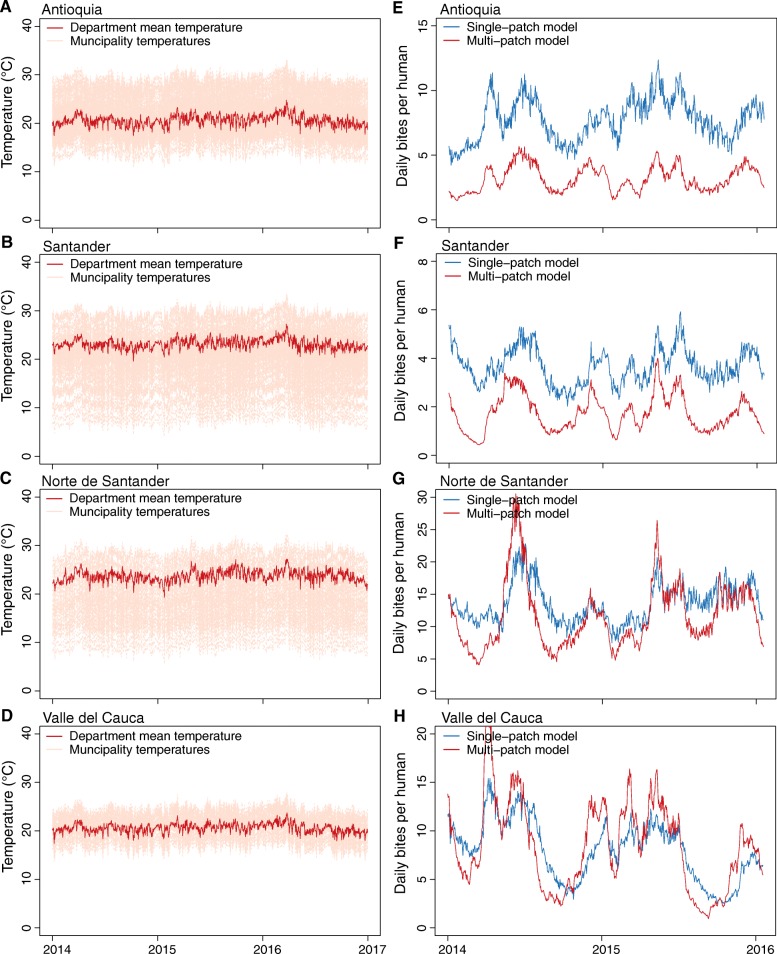
**a**–**d** The population weighted mean daily temperature in the labeled department along with the daily temperatures for each municipality in the department. **e**–**h** The mean daily biting rate from the top 10 simulations for the single-patch and multi-patch models. Panels **a**, **b**, **e**, and **f** are departments where the single-patch model severely overestimated the epidemic size. Panels **c**, **d**, **g**, and **h** are departments where the single-patch model did not overestimate the size of the epidemic

The estimated amount of larval habitat per capita was higher in the single-patch than in the multi-patch model for many of the departments (Additional file 1: Figures S2–S9); particularly for departments where the MASE of the multi-patch departmental model was significantly less than the MASE of the single-patch departmental model. In departments with higher single-patch departmental model MASE values and where the model overestimated epidemic size, the estimated larval habitat decay rates tended to be lower than the estimate from the multi-patch departmental model, which also corresponds to larger mosquito populations in the single-patch departmental models (Fig. 4e, f, Additional file 1: Figures S2–S9). The joint distributions for the parameters that dictate importation timing and magnitude are presented in Additional file 1: Figures S10–S17. Model fits were not overly sensitive to varying the symptomatic or reporting rates, with relative single-patch and multi-patch model fits being qualitatively the same for both lower and higher symptomatic/reporting rates (Additional file 1: Figures S18 and S19). The one exception was the multi-patch departmental model for Antioquia, where the number of reported cases was overestimated with both low and high symptomatic rates, but not at the middle rate used in the main analysis.

### Municipality-level fits

**Fig. 5:**
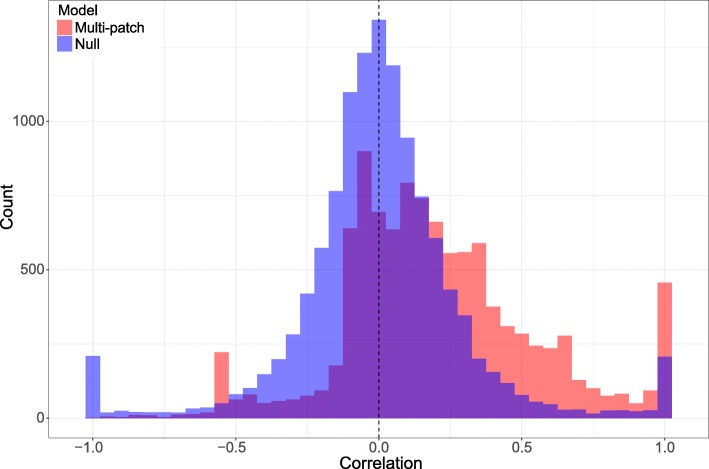
Mean and 95% CI from simulations at the municipality level for Valle del Cauca and Antioquia departments. The four largest municipality-level epidemics for each department are shown

**Fig. 6:**
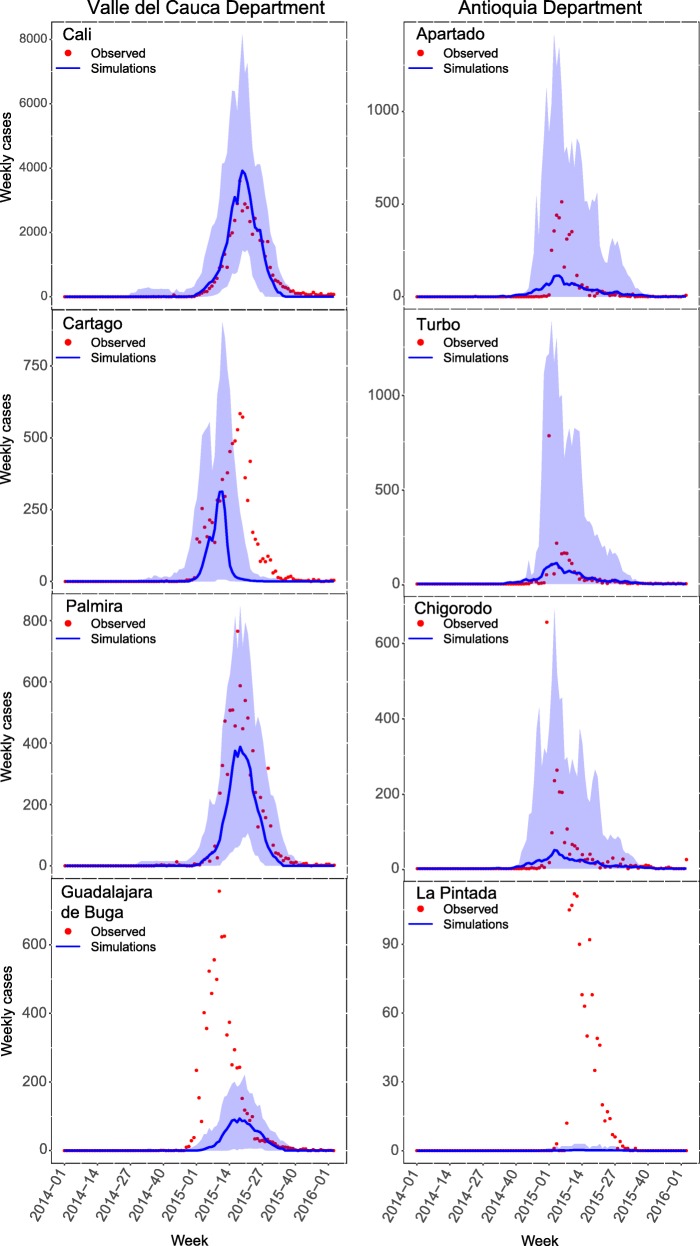
Histogram of correlations (Pearson’s *r*) between the observed and simulated cumulative per capita incidence per municipality. Correlations for the multi-patch departmental models (*red*) and (*blue*) correlations for the null model where departmental cases are allocated to each municipality proportional to its population size

Although the multi-patch simulations for each department were only fitted to the department-level time series, the ensemble of municipality-level simulations captured several important aspects of the observed municipal-level dynamics. Overall, the total number of simulated cases per municipality was strongly correlated with the observed number of cases per municipality (across simulation runs: median *r* = 0.86; interquartile range (IQR) of *r* = 0.53–0.97). At the same time, a null model (in which the single-patch departmental model results were allocated to municipalities proportional to population) produced similar results (median *r* = 0.84; IQR 0.52–0.97). A bigger distinction between the multi-patch and single-patch departmental models was seen when examining per capita incidence. In this case, the correlation between observed and simulated per capita incidence for the multi-patch model (median *r* = 0.17; IQR –0.02 to 0.39) was clearly higher than the single-patch model (median *r* = 0.00; IQR –0.13 to 0.13) (Fig. 5). Whereas the result about raw incidence reflects the importance of population size in driving overall case numbers, the result about per capita incidence demonstrates that there the parameters and assumptions of the multi-patch model contain information about risk not captured by the data to which the model was fitted. Examples of municipality-level estimates are presented in Fig. 6.

## Discussion

At the national level, aggregating simulated epidemics from single-patch departmental models did not improve the estimate of overall epidemic size compared to the single-patch national model fitted directly to national-level data. However, the aggregated single-patch departmental models did improve the shape of the reconstructed national-level epidemic curve, with the timing of the peak correctly estimated in early 2015 instead of several months later by the single-patch national model. This result indicates that the single-patch departmental models were somehow more appropriate for their respective time series than was the single-patch national model for its time series, similar to a previous finding about Zika dynamics in Colombia [61]. This result is particularly concerning for the prospect of using a national-level model for forecasting, due to the fact that it was incapable of capturing the temporal trajectory of the epidemic (fitting early patterns but overestimating later ones). The fact that it could not capture the shape of the epidemic’s trajectory, even under ideal circumstances of being fitted to the entire time series, suggests structural limitations of the model posed at this scale. Two primary limitations are: (1) it does not allow for the timing of the start of the epidemic to vary locally, and (2) it averages spatial covariates over a ludicrously large scale in a country spanning the Andes to the Amazon. Any decisions based on forecasts from such a model could lead to the misallocation of critical resources or undue panic if communicated to the public [62].

Going even further, the collection of multi-patch departmental models also appeared more structurally appropriate for the department-level time series to which they were fitted, meaning that greater spatial granularity in model structure consistently led to improved structural appropriateness for capturing temporal dynamics [21], at least down to the municipal level. In fact, with the multi-patch departmental models, we were able to accurately estimate both the timing and the size of the overall epidemic peak. Both the single-patch and multi-patch departmental models also predicted variability in the national-level time series better than the single-patch national model. Rather than a smooth epidemic curve, there were several noticeable spikes in the national-level time series following the introduction of CHIKV into a new department or large municipality. By estimating introductions into each department, both single-patch and multi-patch departmental models can capture this temporal heterogeneity. The multi-patch departmental model can also simulate introductions at the municipality level, allowing for exploration of which municipalities might have been the most likely entry point for a given department. In general, our results raise concerns about the application of national-level models to national-level time series, as has been done previously for the chikungunya invasion of the Americas [63, 64]. It is essential that population substructure be included in models fitted to national-level data, and our multi-patch model represents a structurally advantageous option, as do others (e.g., [16]).

With respect to departmental dynamics, two major patterns emerged when we compared the relative fits of the single-patch and multi-patch departmental models. First, for many of the departments where the relative MASE of the multi-patch model was substantially lower, the single-patch model provided a poorer fit to the observed data because it overestimated the size of the epidemic (e.g., Antioquia, Atlantico, Risaralda, and Santander). In these departments, the single-patch model may have overestimated the amount of available larval mosquito habitat, or estimated a slower decay in larval habitat size following rainfall. Because the climate variables were averaged for the entire department, the mean temperature in many departments was less suitable for *Ae. aegypti* and CHIKV transmission than it was in some of the municipalities within the department (Fig. 4a–d). This may be especially true for a mountainous country such as Colombia, consistent with general expectations that the nature of spatial autocorrelation affects the type of bias that results from spatial aggregation [65]. If climate suitability is lower, then more larval habitat is needed to achieve the same number of infectious mosquitoes per human (Fig. 4e–h). Because the entire department is homogeneously mixed, everyone in the department experiences a similar risk of infection, and the size of the epidemic is overestimated. The multi-patch models, however, may contain municipalities where the climate is not suitable for efficient CHIKV transmission, lowering the portion of the population at risk of infection and appropriately matching geographic variation in human demography with geographic variation in climate. This issue of appropriately matching populations with factors driving exposure is a general and pervasive issue in spatial epidemiology, affecting not only vector-borne diseases but even non-communicable diseases such as leukemia [66].

The second major pattern was displayed by single-patch departmental models where the timing of the peak and the final epidemic size fit relatively well, but the duration of the epidemic was underestimated. In these departments (e.g., Huila, Meta, and Tolima), the single-patch model overestimated the initial increase in cases at the start of the epidemic, and then underestimated how long it would take for the epidemic to fade out after the peak. The multi-patch model may have done a better job of estimating the rapid increase in cases at the start of the epidemic because the conditions in one or more municipalities were highly suitable for rapid transmission compared to mean climate conditions across the department. Once the peak was reached, these departments could also experience a slower decline in cases because municipalities with less favorable conditions would take longer to reach their local peaks. In addition, the spatial structuring of the human population and movement within a structured population slows the spread of the epidemic within the department [67]. These results mirror recent work [68] on influenza dynamics made possible by fine-scale spatial data, which showed that a combination of detailed human geographic data and mobility patterns is important for being able to recreate spatially heterogeneous epidemic patterns below larger scales of spatial aggregation.

No single pattern or set of patterns was observed in departments where the multi-patch model did not improve on the fit of the single-patch departmental model. In several departments, such as Bolivar and Norte de Santander, the single-patch departmental model provided a good fit to the data, leaving little room for improvement with the multi-patch model. There were several departments with smaller outbreaks, particularly Boyaca and Nariño, where the multi-patch rather than the single-patch departmental model had a tendency to overestimate the size of the epidemic. For both of these departments, the mean estimate from the multi-patch departmental model was actually a better fit, but the variance between simulations was greater, likely due to the additional stochasticity that arises from the possibility of stochastic fadeout occurring in each municipality in a multi-patch model. There were also several departments with smaller population sizes that had relative MASE scores near one. These departments, such as Amazonas and Vaupes, had few cases, and as a result neither the single-patch nor the multi-patch models estimated that an outbreak had occurred.

Impressively, our assumptions about transmission dynamics within and among municipalities turned out to be good enough to enable estimation, to at least some degree, of per capita incidence below the spatial scale of the data to which the model was fitted. Implicitly, the single-patch departmental model assumes that residents of all municipalities within a department experience equal risk of infection. Not surprisingly, there was variation in risk among residents of different municipalities, and our multi-patch departmental model provided estimates of that risk that were positively correlated with per capita incidence based on suspected case numbers. Because no data below the departmental scale were used to inform those estimates, this result provides a clear indication that the parameters and assumptions of the multi-patch departmental model contain some degree of positive predictive value. Models of mosquito-borne pathogen transmission usually ignore within-patch heterogeneity [20] and instead default to assuming well-mixed interactions at whatever scale data are available. Our results suggest that this may often be a mistake, given the potential for copious high-resolution data on spatial drivers of transmission [56] and an improved understanding of human mobility patterns [57] to enable successful model predictions at finer scales than that at which data are available. Although gravity models are often capable of reproducing patterns of epidemic spread similar to alternative models of human movement [69], incorporating human movement data from sources such as cell phone metadata can improve model estimates of spread and timing compared to a gravity model [32]. Human movement data or transportation infrastructure information may be particularly useful for modeling epidemic spread in geographically diverse countries like Colombia, where the distance between locations may not be representative of their connectivity due to intervening mountain ranges or rainforests that restrict human movement.

Although the EMOD-DTK modeling framework is flexible in many respects, we madeseveral simplifications that could be viewed as limitations of this study. First, while the 1122 municipalities do represent a granular view of the country, there may be relevant heterogeneities at even finer spatial scales. Dengue spatial foci have been estimated to occur at neighborhood scales [70, 71], and both blood-feeding and microclimate heterogeneity have been shown as far down as the household scale [30, 72]. Theoretical results indicate that these extremely fine-scale heterogeneities may not be easily captured by even modestly aggregated models [21]. Second, we assumed a single, homogeneous larval mosquito habitat for each municipality within a department. In reality, these habitats are extremely numerous [73] and are spatially associated with many factors [74]. More detailed models of *Ae. aegypti* population dynamics exist [75], but they come at exceedingly high computational expense for the spatial scales of interest here and are subject to numerous uncertainties [76]. Still, different models of *Ae. aegypti* population dynamics can vary considerably in their response to climatic drivers and interventions [77], suggesting that future refinement of this aspect of the model may be worthwhile. Third, besides climate, there are other important factors that influence geographic heterogeneity in incidence rates that we did not incorporate into our model that could improve estimates at the department or municipality level. One important factor that is known to influence both the amount of mosquito habitat and human contact with mosquitoes is the local level of economic development, with poorer areas having higher incidence rates due to higher contact rates with *Aedes* mosquitoes [78]. Other environmental factors might also affect the local suitability for larval mosquitoes, such as how local infrastructure and development, as well as cultural practices surrounding water storage, influence the amount of mosquito breeding habitat. Fourth, we assumed a fixed reporting rate based on an estimate for chikungunya from Puerto Rico and overall estimates for dengue, although reporting rates are likely to vary among departments or even among municipalities [79].

## Conclusions

Simulating CHIKV transmission dynamics from versions of our model with increasing spatial granularity improved the fit of the model to temporal incidence patterns, both at the scales to which the data were fitted and when aggregated at the national level. This improvement derived from the fact that simulations with spatially granular models more appropriately captured spatial heterogeneity in epidemiologically relevant factors, such as mosquito abundance and human demography and movement. This improvement was evident when moving from national to departmental levels and from departmental to municipal levels. Models based on municipal-level spatial heterogeneity closely matched epidemic size for the majority of departments and also estimated the duration of the epidemic better than the single-patch departmental models, particularly with respect to the timing of the start of local epidemics. These models also captured continued low levels of transmission for months following epidemic peaks in many of the departments. Use of models posed at spatial scales more granular than those at which data are available represents a promising approach for the common situation of needing to answer questions about spatial heterogeneity in transmission below the scale at which highly spatially aggregated data are available.

## Additional file

Additional file 1: **Figure S1.** (A) Cumulative incidence as a function of the maximum adaptive sampling population size. *Dashed line* represents the mean, and the *dotted lines* are the mean ± the standard deviation. (B) Epidemic time series for three different maximum adaptive sampling population sizes. *Solid lines* are means *and shaded areas* represent the range. **Figures S2**–**S9.** The joint distribution of parameter estimates for amount of rainfall-associated temporary larval mosquito habitat and the decay rate of that temporary habitat. *Left panels* are estimates from the single-patch departmental model, and *right panels* are estimated from the multi-patch departmental model. Each figure contains results from four departments, with the departments ordered from lowest to highest relative MASE as displayed in Fig. 2. **Figures S10**–**S17.** The joint distribution of parameter estimates for the timing of the initial importation event(s) and the magnitude of importation. *Left panels* are estimates from the single-patch departmental model, and *right panels* are estimated from the multi-patch departmental model. Each figure contains results from four departments, with the departments ordered from lowest to highest relative MASE as displayed in Fig. 2. **Figures S18**–**S19.** Comparisons of department-level results for single-patch and multi-patch models for three different symptomatic rates (0.54, 0.72, and 0.90). *Black dots* represent the observed time series, *darker colored lines* are the single best-fitting simulations, and *lighter colored lines* are the other 40 top simulations. (PDF 40161 kb)

## Abbreviations

CHIKV: Chikungunya virus
MASE: Mean absolute scaled error
