# Supplementary material for: Local and regional dynamics of chikungunya virus transmission in Colombia: the role of mismatched spatial heterogeneity
Source: BMC Med. 2018 Aug 30;16:152. doi: 10.1186/s12916-018-1127-2 (PMC6116375; doi:10.1186/s12916-018-1127-2)
Supplement: Supplementary file 1 — Figure S1. (A) Cumulative incidence as a function of the maximum adaptive sampling population size. Dashed line represents the mean, and the dotted lines are the mean ± the standard deviation. (B) Epidemic time series for three different maximum adaptive sampling population sizes. Solid lines are means and shaded areas represent the range. Figures S2–S9. The joint distribution of parameter estimates for amount of rainfall-associated temporary larval mosquito habitat and the decay rate of that temporary habitat. Left panels are estimates from the single-patch departmental model, and right panels are estimated from the multi-patch departmental model. Each figure contains results from four departments, with the departments ordered from lowest to highest relative MASE as displayed in Fig. 2. Figures S10–S17. The joint distribution of parameter estimates for the timing of the initial importation event(s) and the magnitude of importation. Left panels are estimates from the single-patch departmental model, and right panels are estimated from the multi-patch departmental model. Each figure contains results from four departments, with the departments ordered from lowest to highest relative MASE as displayed in Fig. 2. Figures S18–S19. Comparisons of department-level results for single-patch and multi-patch models for three different symptomatic rates (0.54, 0.72, and 0.90). Black dots represent the observed time series, darker colored lines are the single best-fitting simulations, and lighter colored lines are the other 40 top simulations. (PDF 40161 kb) [file 12916_2018_1127_MOESM1_ESM.pdf]

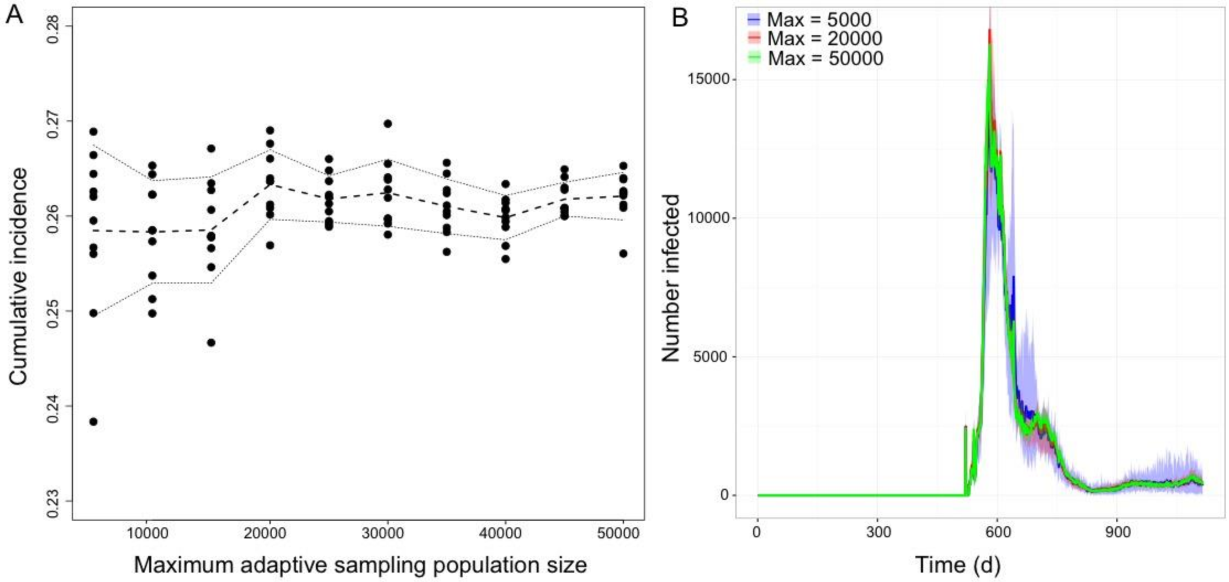

Figure S1. (A) Cumulative incidence as a function of the maximum adaptive sampling population size. Dashed line represents the mean and the dotted lines are the mean ± the standard deviation. (B) Epidemic time series for three different maximum adaptive sampling population sizes. Solid lines are means and shaded areas represent the range.

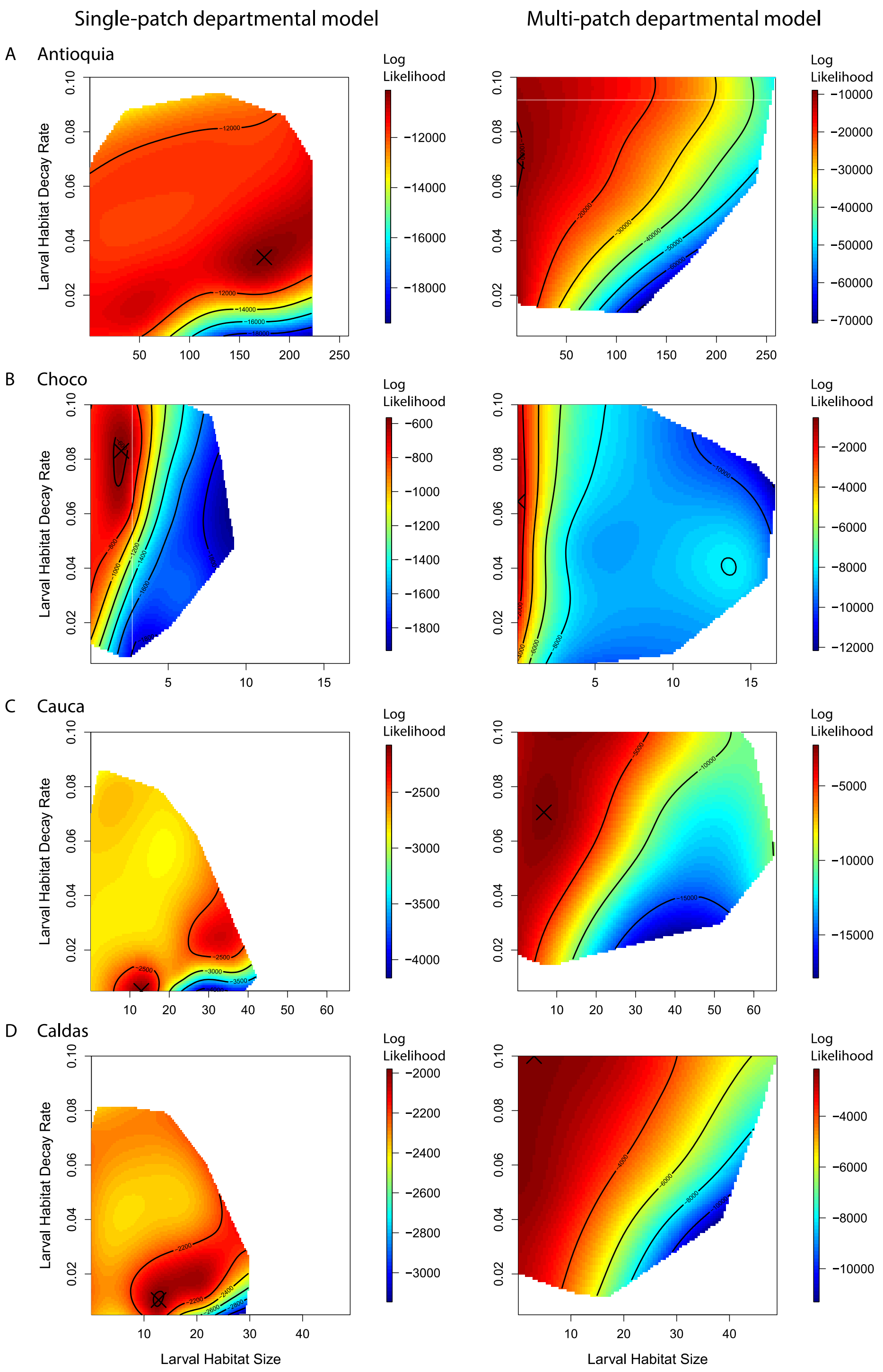

Figure S2. The joint distribution of parameter estimates for amount of rainfall-associated temporary larval mosquito habitat and the decay rate of that temporary habitat. Left panels are estimates from the single-patch departmental model and right panels are estimated from the multi-patch departmental model. Each figure contains results from four departments, with the departments order from lowest to highest relative MASE as displayed in Figure 2.

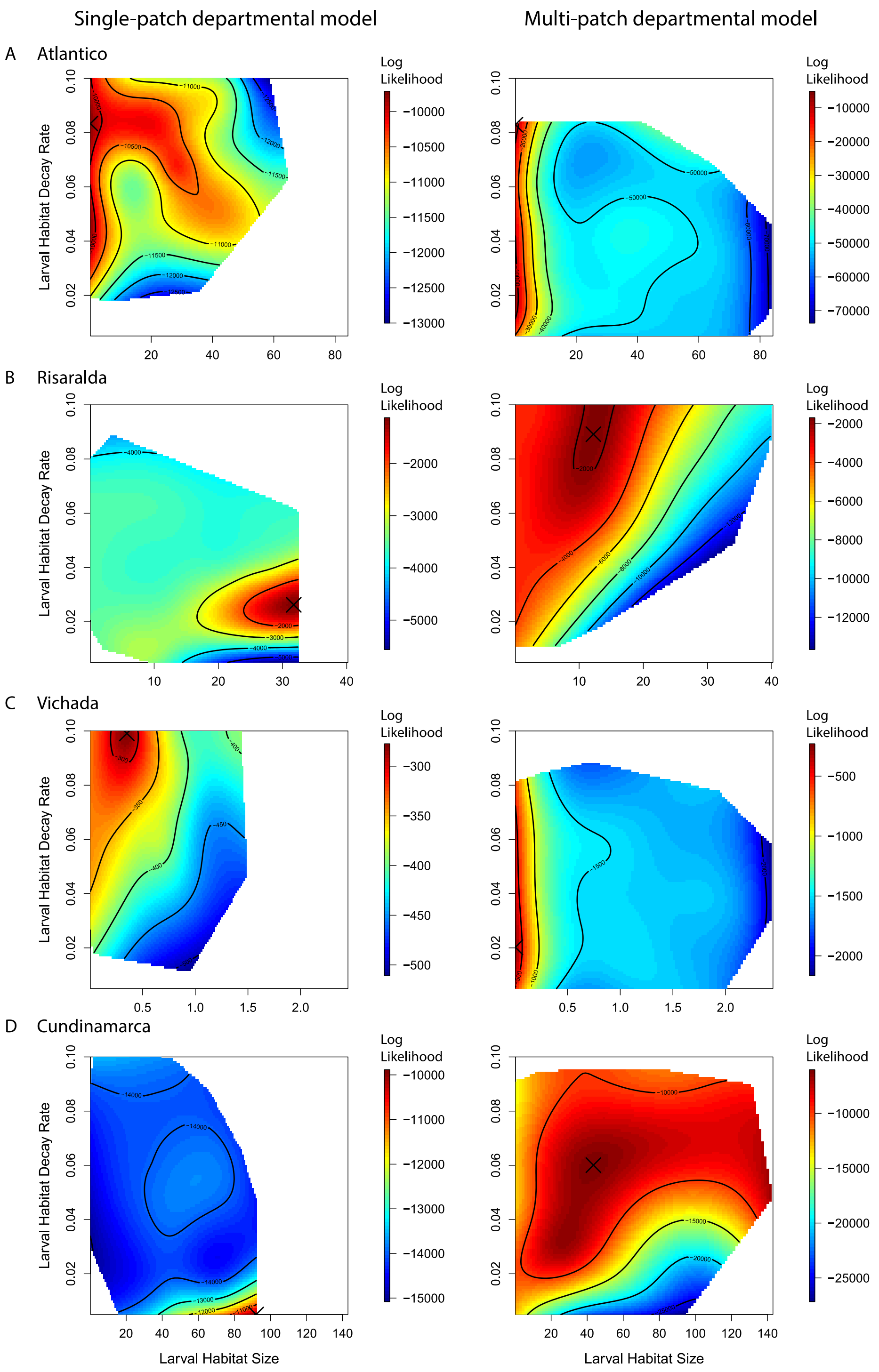

Figure S3. The joint distribution of parameter estimates for amount of rainfall-associated temporary larval mosquito habitat and the decay rate of that temporary habitat. Left panels are estimates from the single-patch departmental model and right panels are estimated from the multi-patch departmental model. Each figure contains results from four departments, with the departments order from lowest to highest relative MASE as displayed in Figure 2.

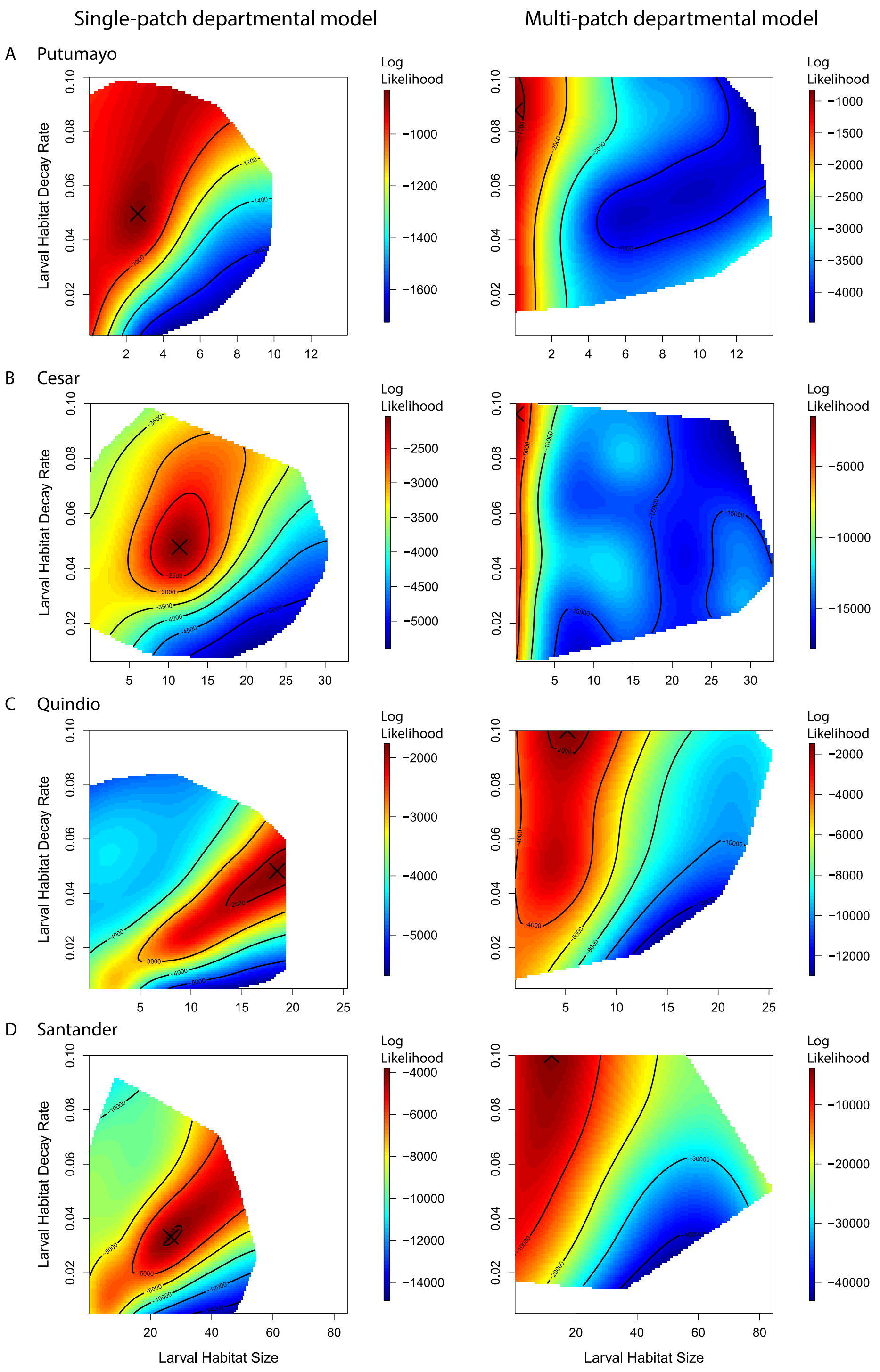

Figure S4. The joint distribution of parameter estimates for amount of rainfall-associated temporary larval mosquito habitat and the decay rate of that temporary habitat. Left panels are estimates from the single-patch departmental model and right panels are estimated from the multi-patch departmental model. Each figure contains results from four departments, with the departments order from lowest to highest relative MASE as displayed in Figure 2.

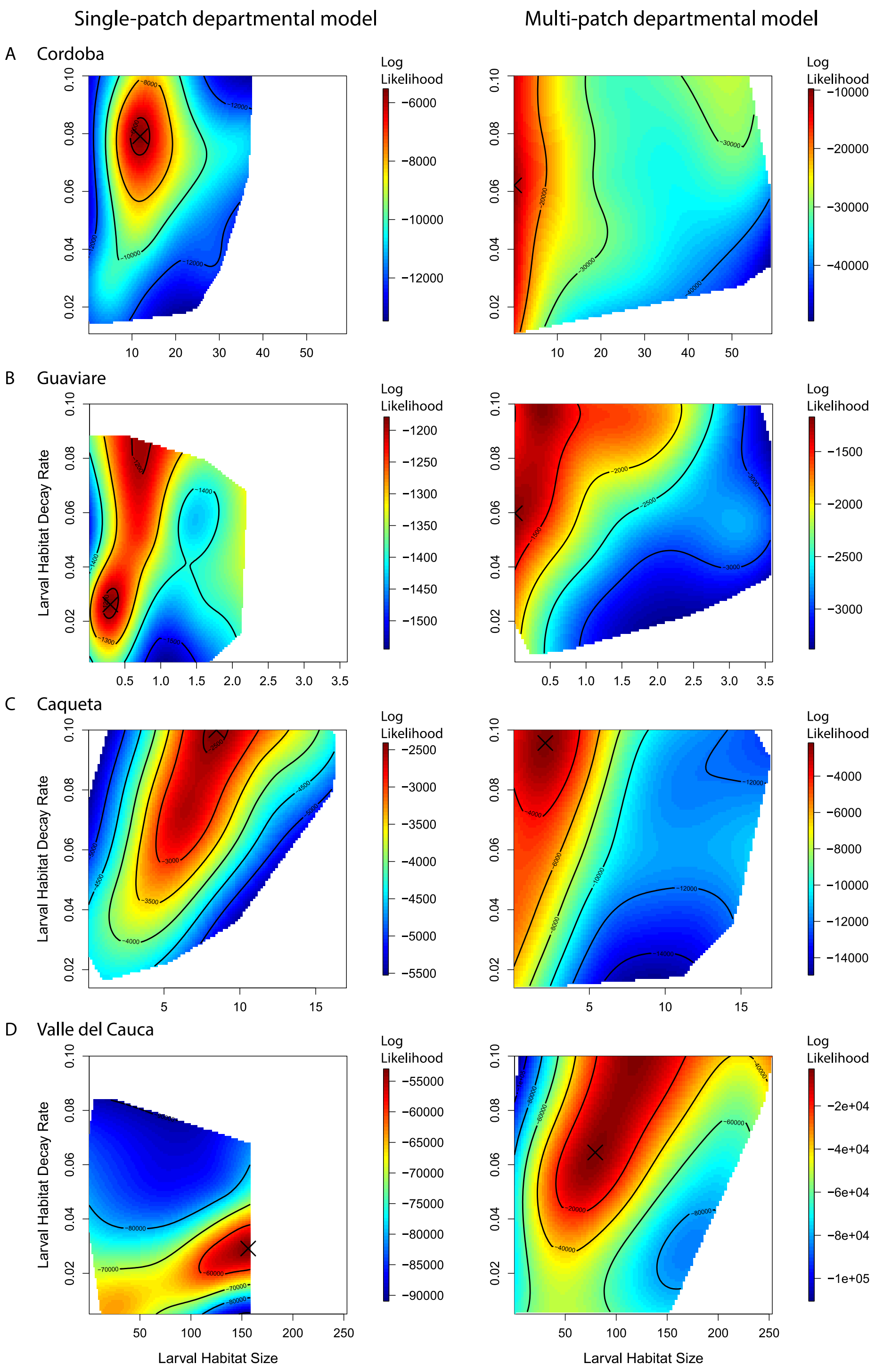

Figure S5. The joint distribution of parameter estimates for amount of rainfall-associated temporary larval mosquito habitat and the decay rate of that temporary habitat. Left panels are estimates from the single-patch departmental model and right panels are estimated from the multi-patch departmental model. Each figure contains results from four departments, with the departments order from lowest to highest relative MASE as displayed in Figure 2.

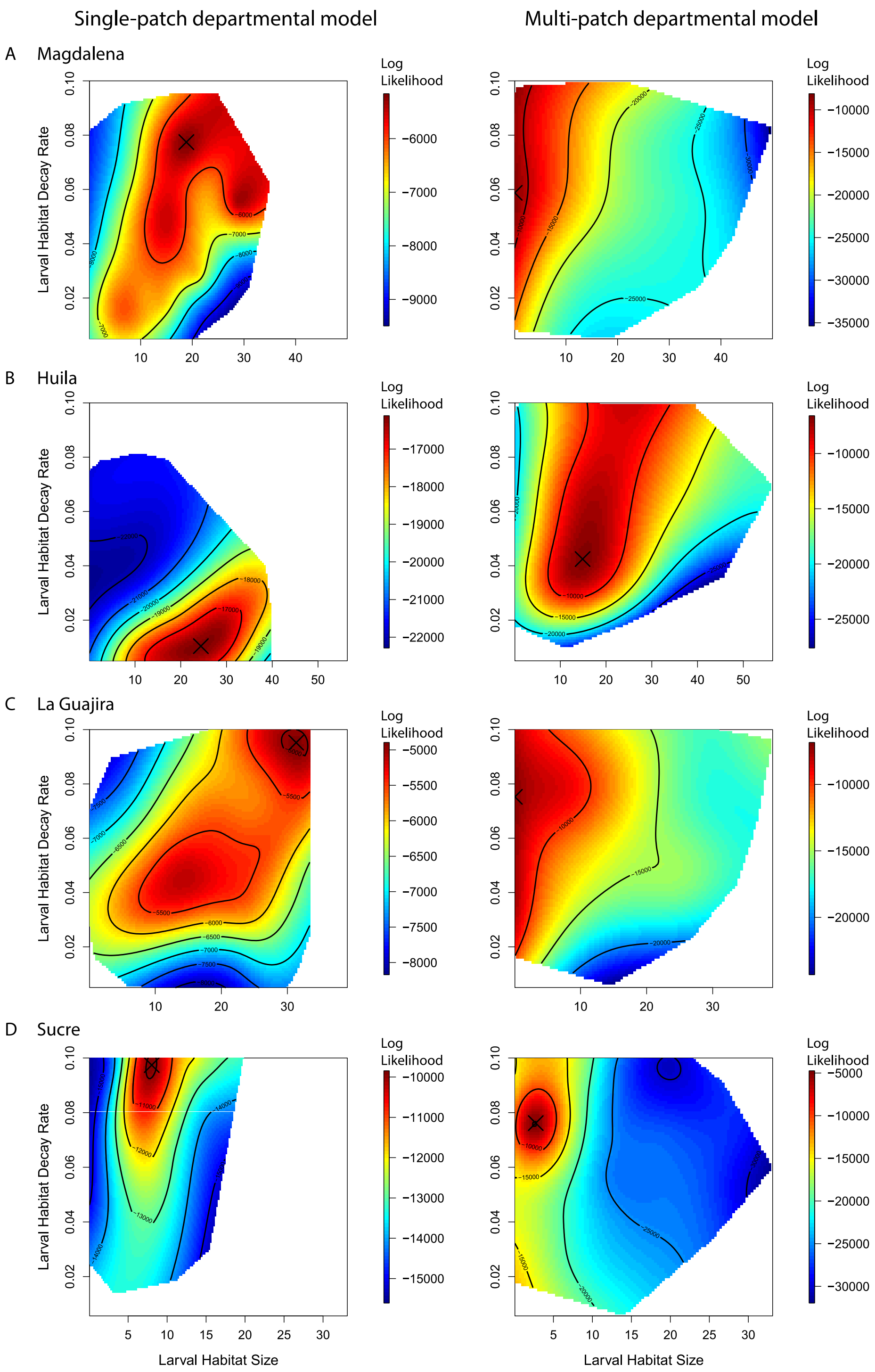

Figure S6. The joint distribution of parameter estimates for amount of rainfall-associated temporary larval mosquito habitat and the decay rate of that temporary habitat. Left panels are estimates from the single-patch departmental model and right panels are estimated from the multi-patch departmental model. Each figure contains results from four departments, with the departments order from lowest to highest relative MASE as displayed in Figure 2.

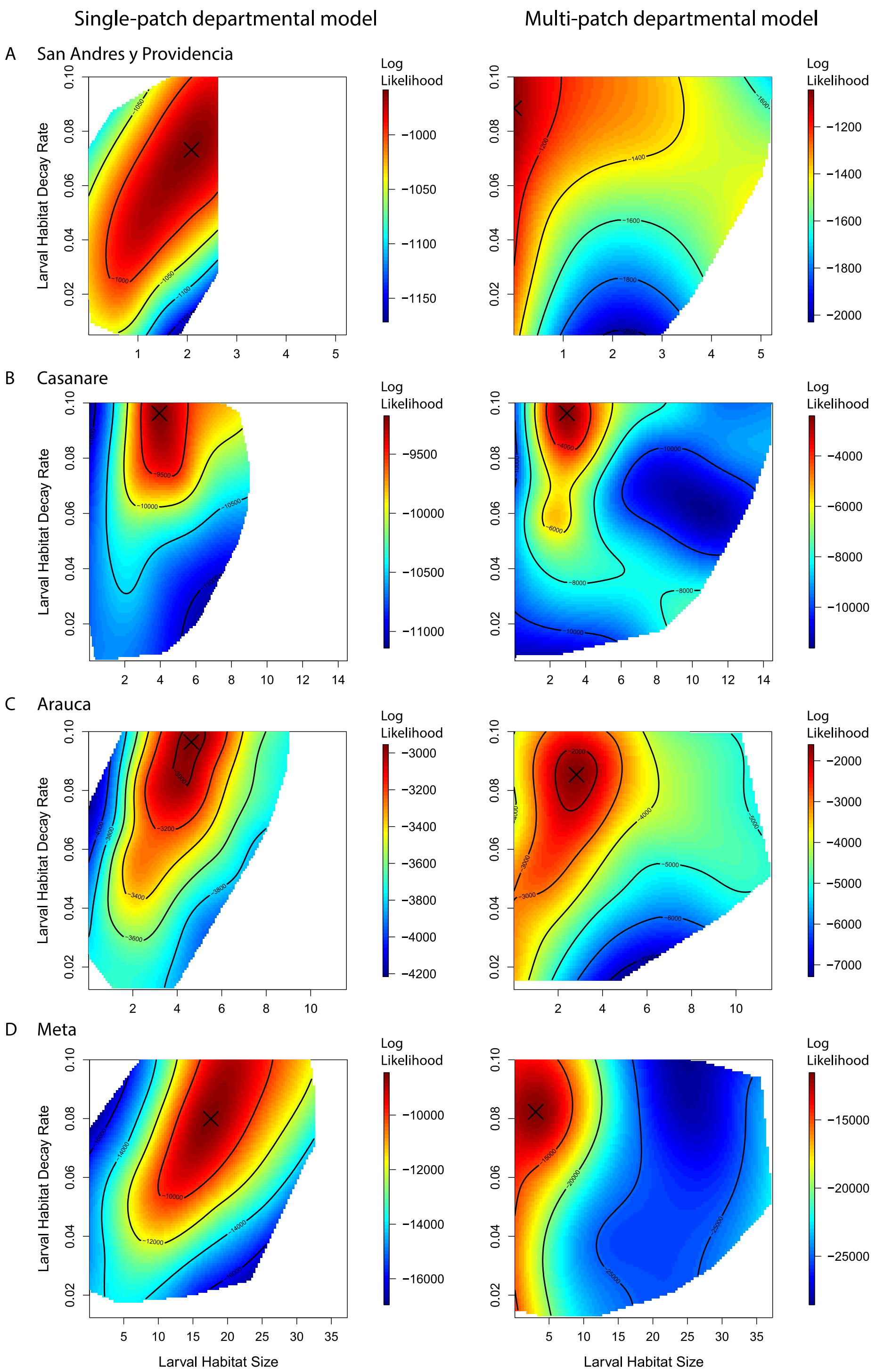

Figure S7. The joint distribution of parameter estimates for amount of rainfall-associated temporary larval mosquito habitat and the decay rate of that temporary habitat. Left panels are estimates from the single-patch departmental model and right panels are estimated from the multi-patch departmental model. Each figure contains results from four departments, with the departments order from lowest to highest relative MASE as displayed in Figure 2.

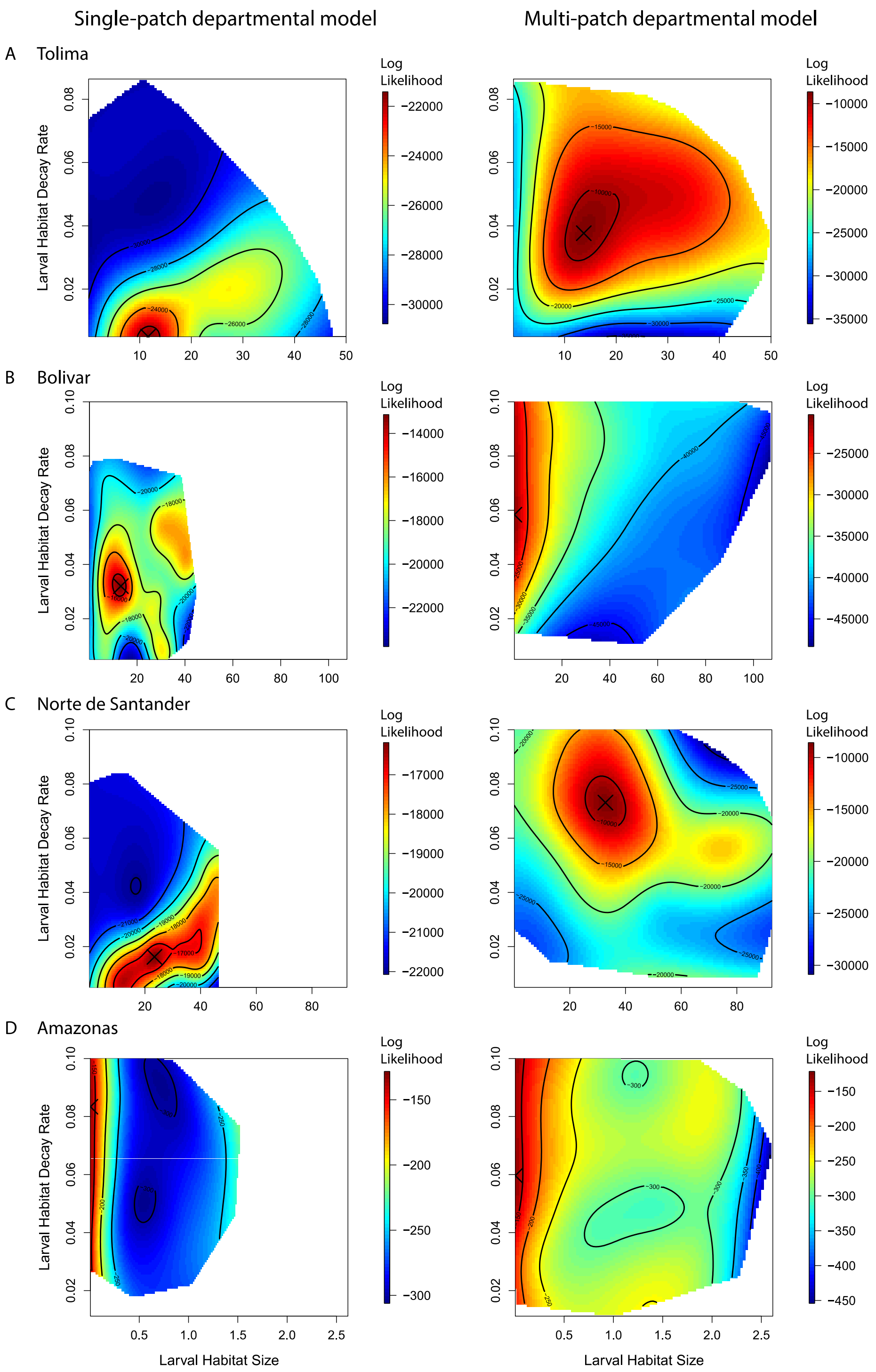

Figure S8. The joint distribution of parameter estimates for amount of rainfall-associated temporary larval mosquito habitat and the decay rate of that temporary habitat. Left panels are estimates from the single-patch departmental model and right panels are estimated from the multi-patch departmental model. Each figure contains results from four departments, with the departments order from lowest to highest relative MASE as displayed in Figure 2.

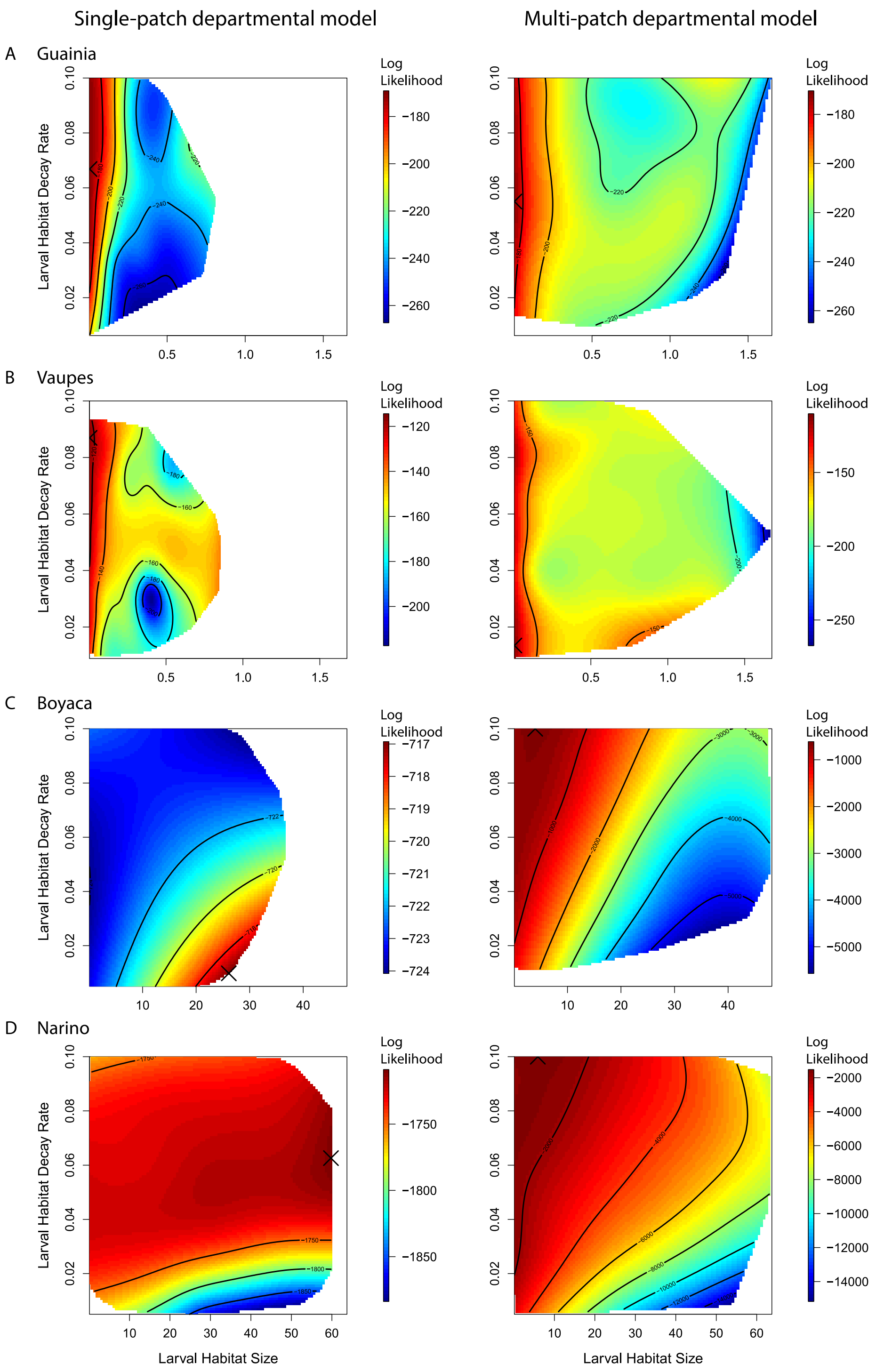

Figure S9. The joint distribution of parameter estimates for amount of rainfall-associated temporary larval mosquito habitat and the decay rate of that temporary habitat. Left panels are estimates from the single-patch departmental model and right panels are estimated from the multi-patch departmental model. Each figure contains results from four departments, with the departments order from lowest to highest relative MASE as displayed in Figure 2.

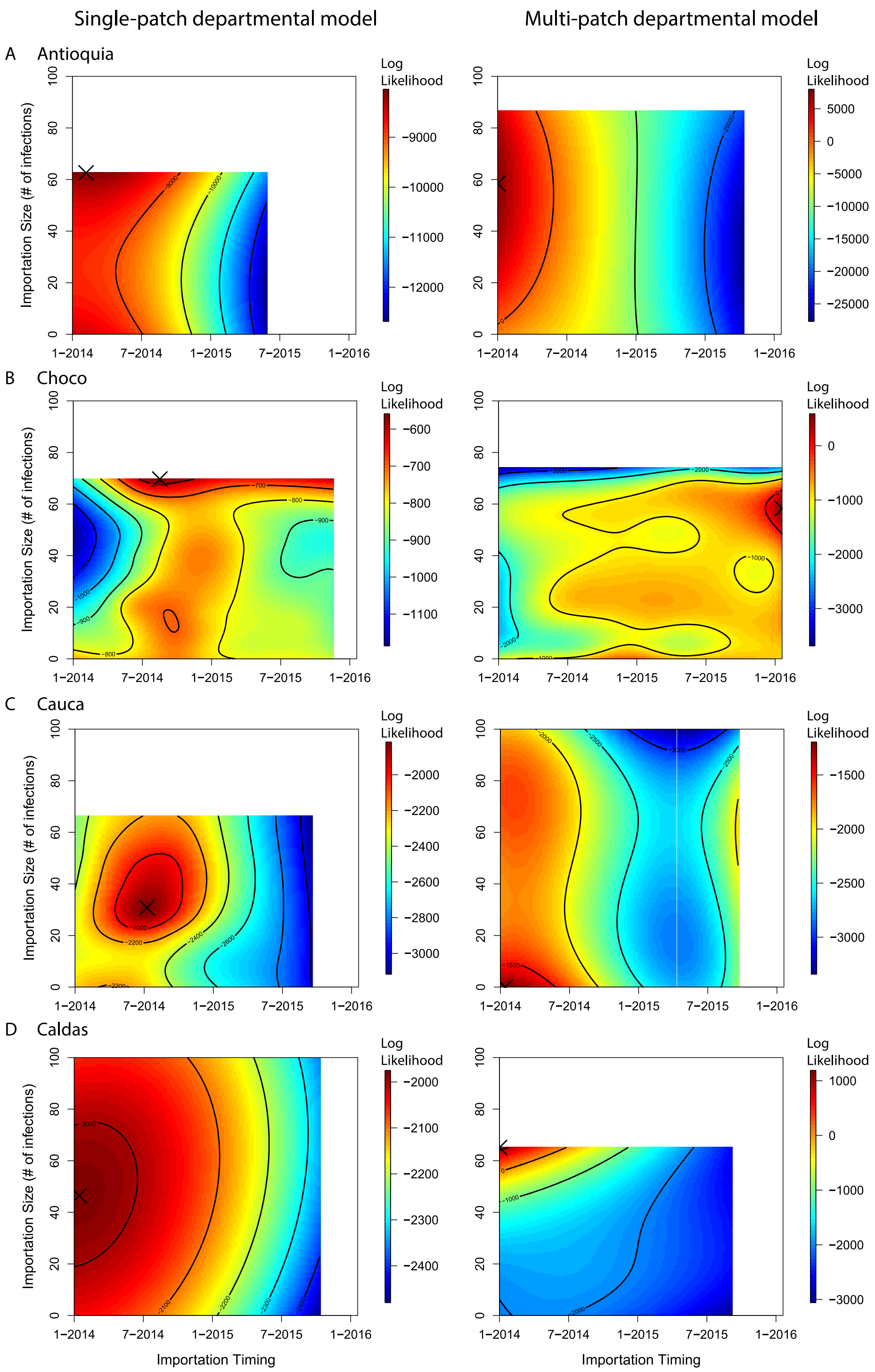

Figure S10. The joint distribution of parameter estimates for the timing of the initial importation event(s) and the magnitude of importation. Left panels are estimates from the single-patch departmental model and right panels are estimated from the multi-patch departmental model. Each figure contains results from four departments, with the departments order from lowest to highest relative MASE as displayed in Figure 2.

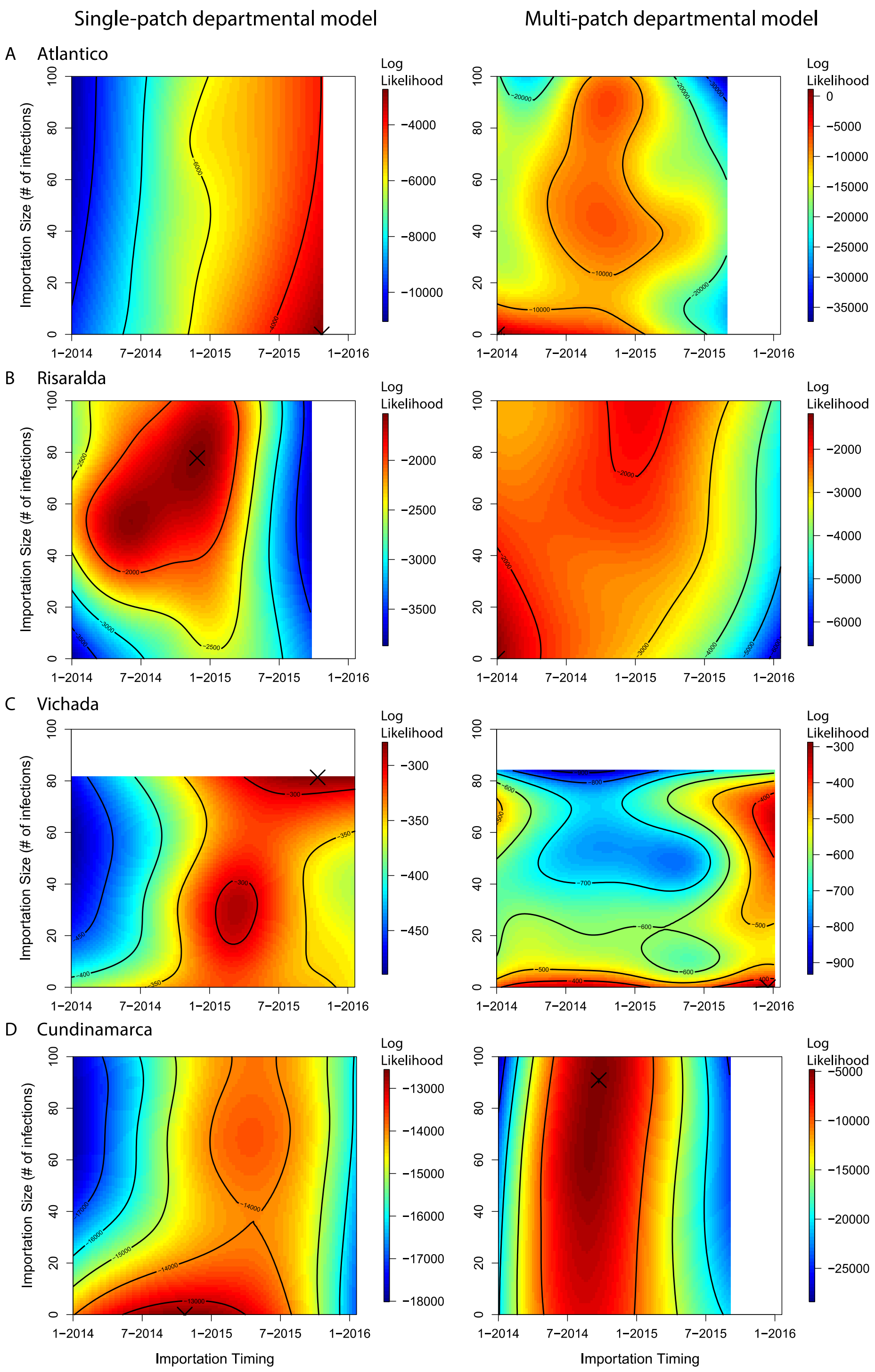

Figure S11. The joint distribution of parameter estimates for the timing of the initial importation event(s) and the magnitude of importation. Left panels are estimates from the single-patch departmental model and right panels are estimated from the multi-patch departmental model. Each figure contains results from four departments, with the departments order from lowest to highest relative MASE as displayed in Figure 2.

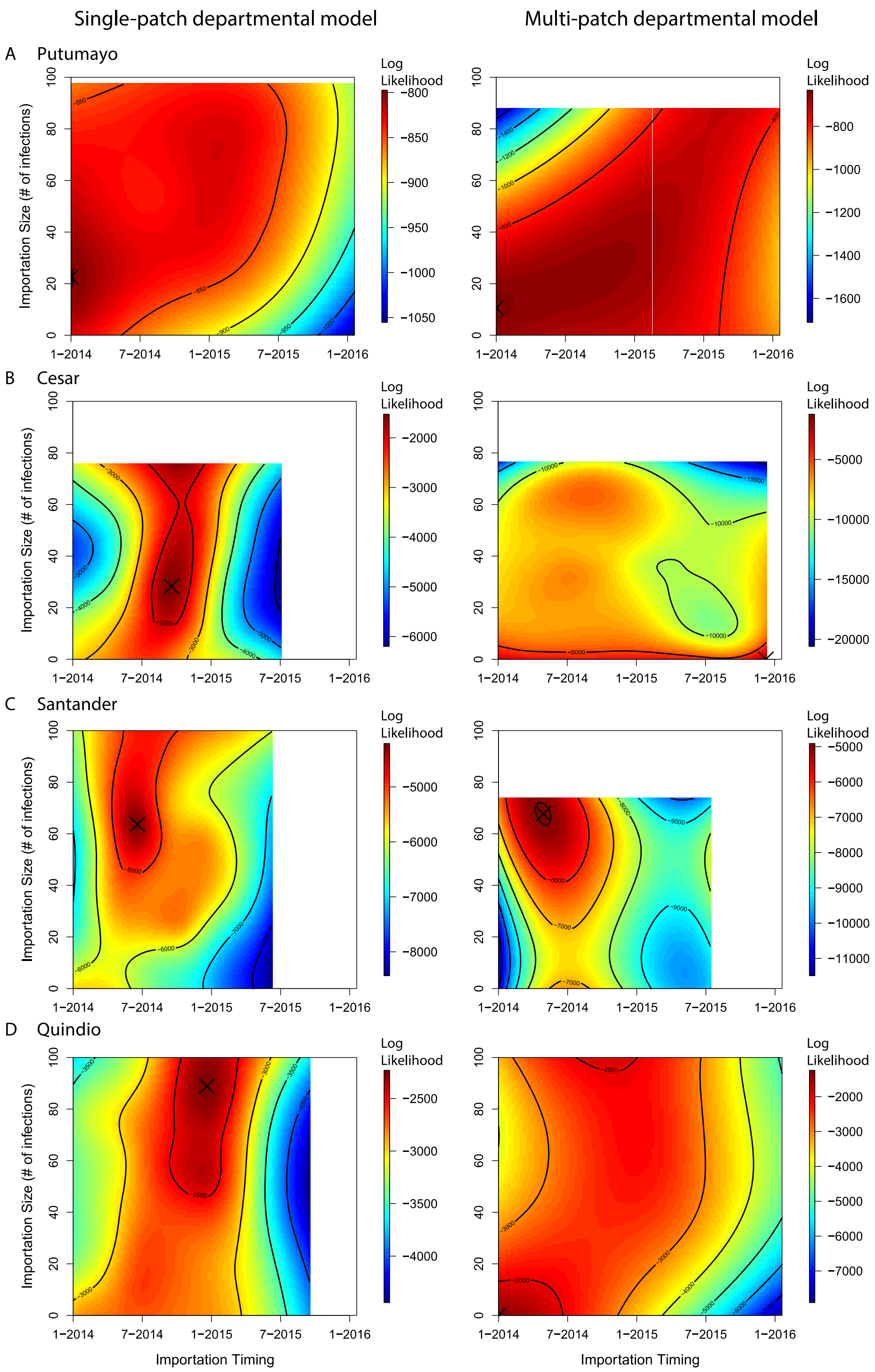

Figure S12. The joint distribution of parameter estimates for the timing of the initial importation event(s) and the magnitude of importation. Left panels are estimates from the single-patch departmental model and right panels are estimated from the multi-patch departmental model. Each figure contains results from four departments, with the departments order from lowest to highest relative MASE as displayed in Figure 2.

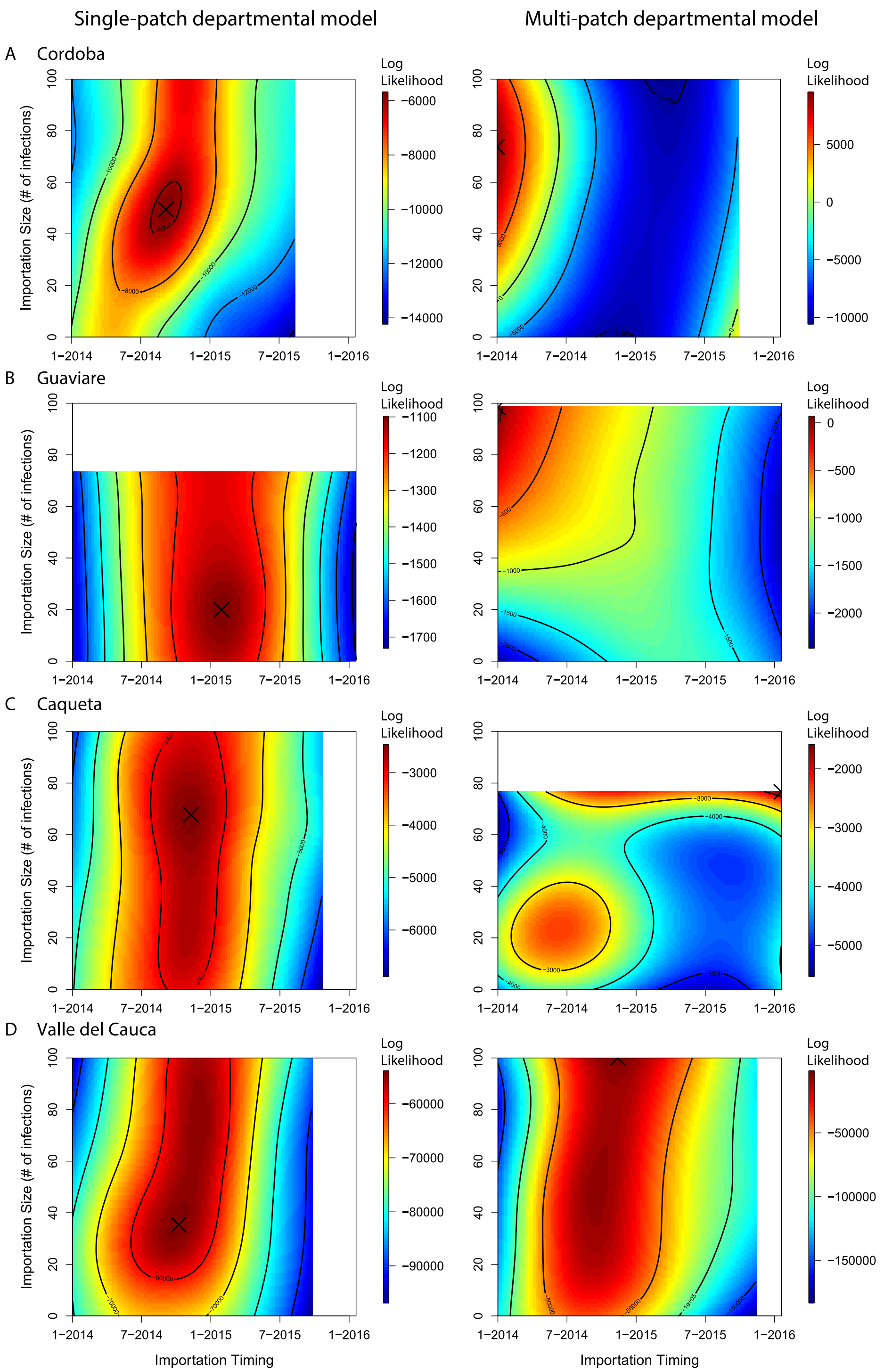

Figure S13. The joint distribution of parameter estimates for the timing of the initial importation event(s) and the magnitude of importation. Left panels are estimates from the single-patch departmental model and right panels are estimated from the multi-patch departmental model. Each figure contains results from four departments, with the departments order from lowest to highest relative MASE as displayed in Figure 2.

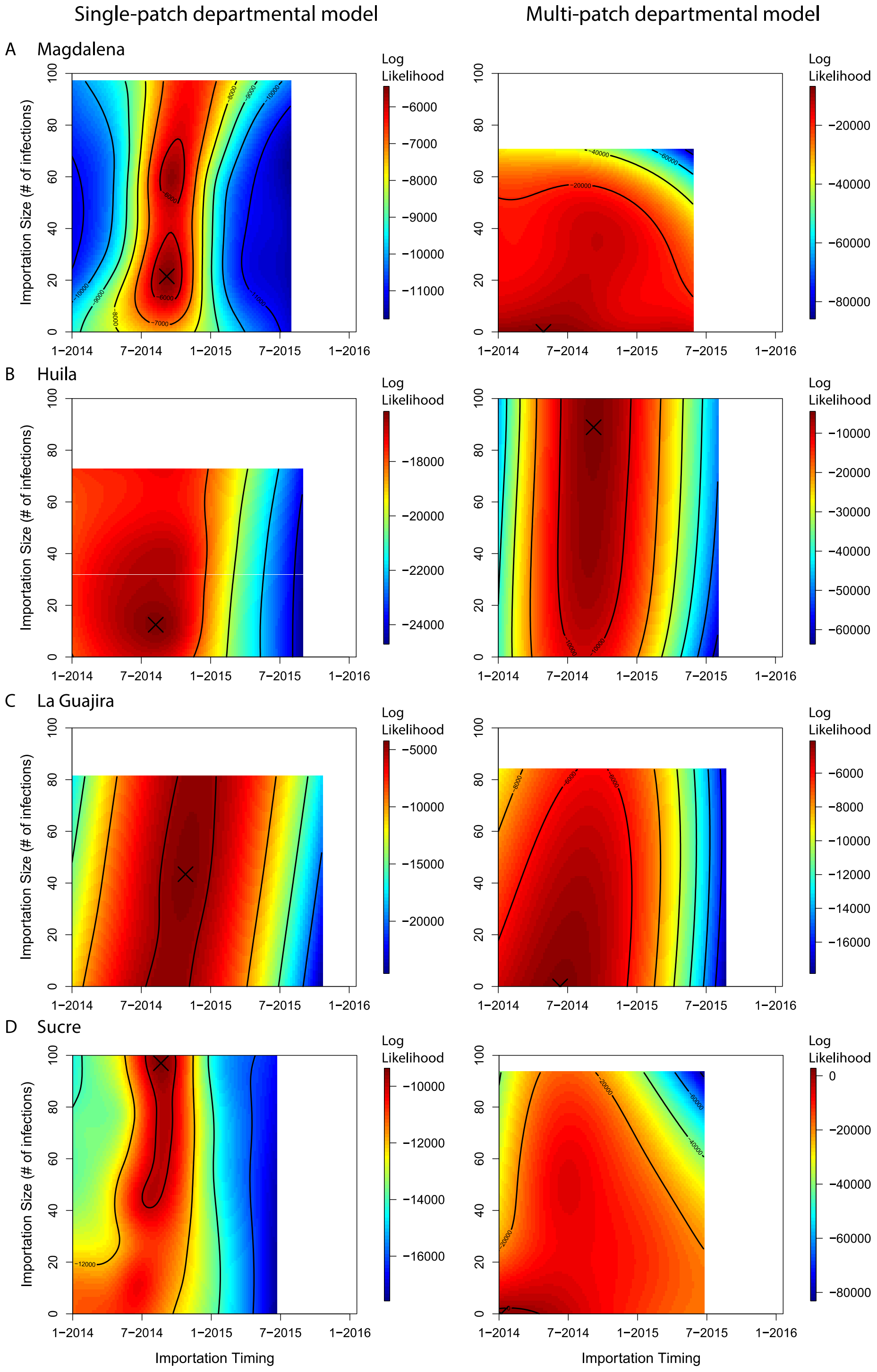

Figure S14. The joint distribution of parameter estimates for the timing of the initial importation event(s) and the magnitude of importation. Left panels are estimates from the single-patch departmental model and right panels are estimated from the multi-patch departmental model. Each figure contains results from four departments, with the departments order from lowest to highest relative MASE as displayed in Figure 2.

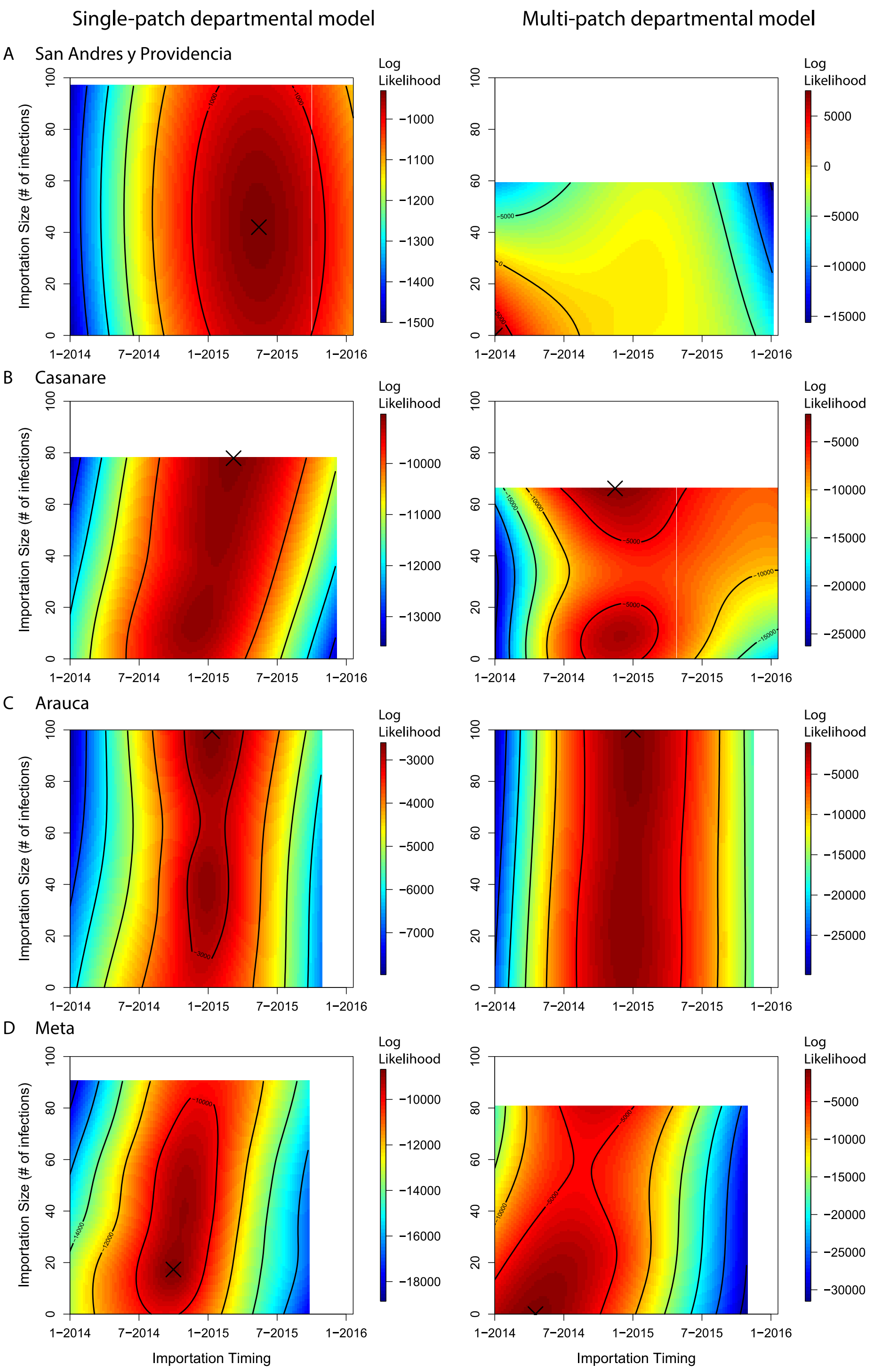

Figure S15. The joint distribution of parameter estimates for the timing of the initial importation event(s) and the magnitude of importation. Left panels are estimates from the single-patch departmental model and right panels are estimated from the multi-patch departmental model. Each figure contains results from four departments, with the departments order from lowest to highest relative MASE as displayed in Figure 2.

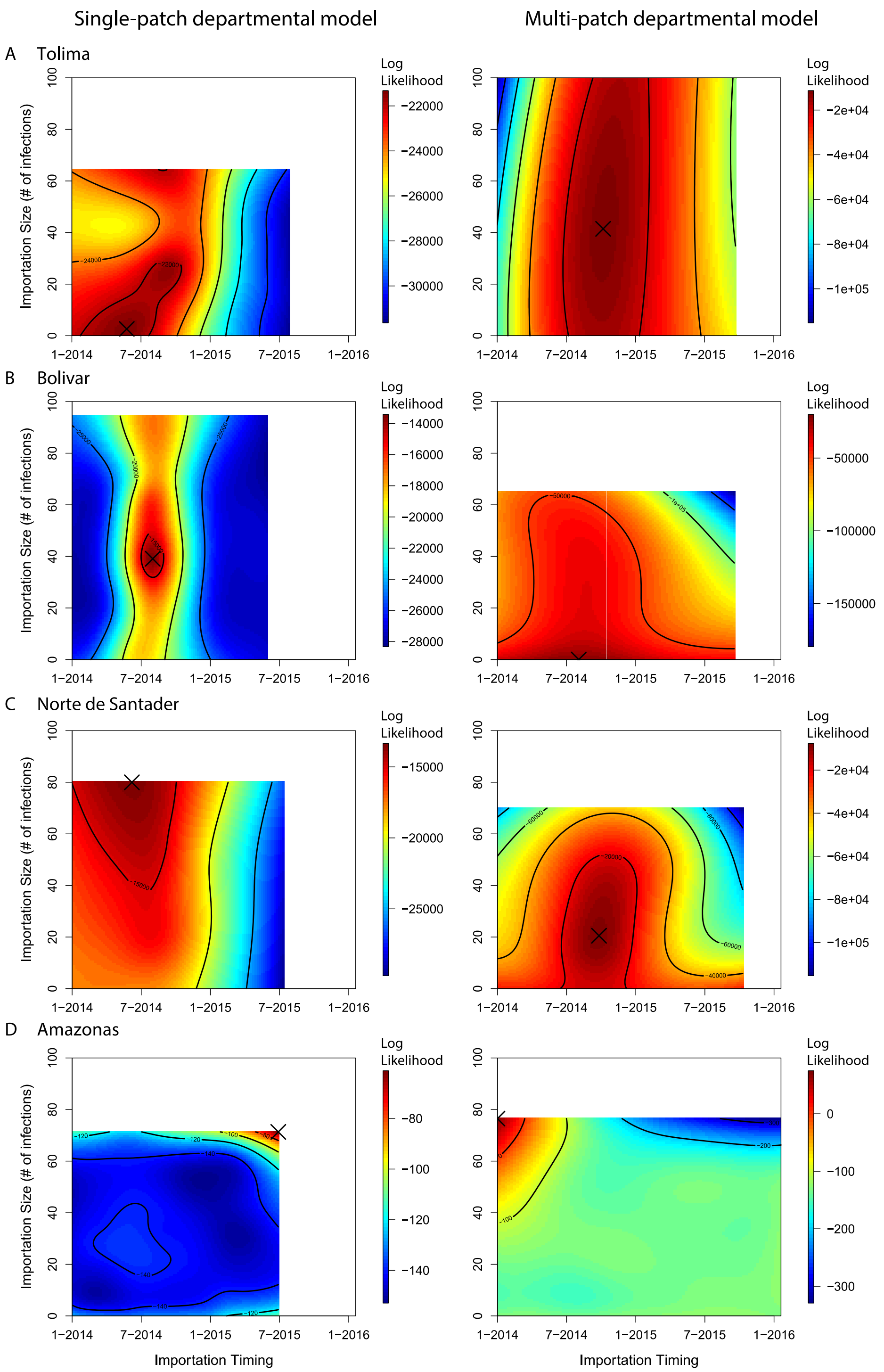

Figure S16. The joint distribution of parameter estimates for the timing of the initial importation event(s) and the magnitude of importation. Left panels are estimates from the single-patch departmental model and right panels are estimated from the multi-patch departmental model. Each figure contains results from four departments, with the departments order from lowest to highest relative MASE as displayed in Figure 2.

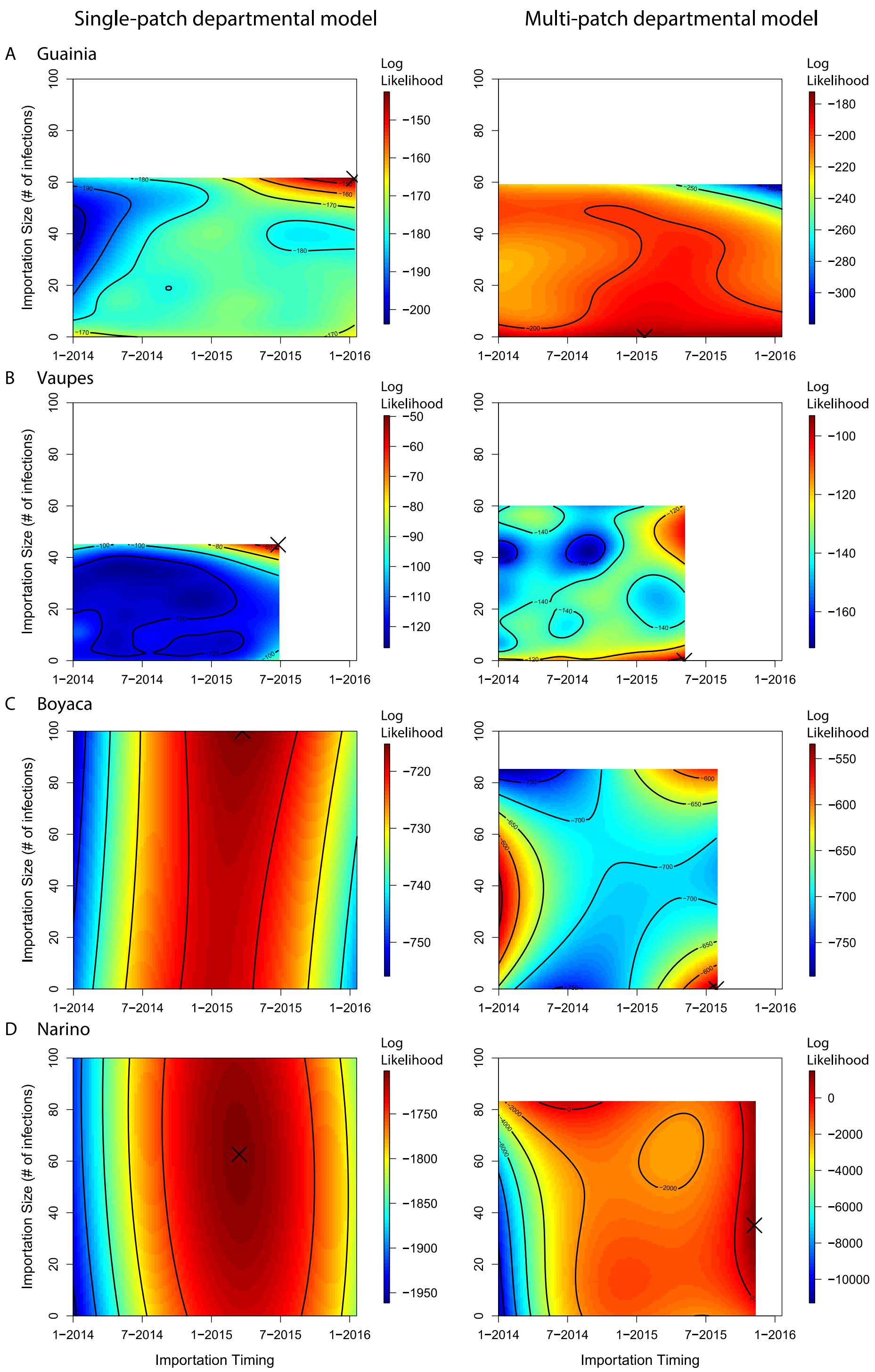

Figure S17. The joint distribution of parameter estimates for the timing of the initial importation event(s) and the magnitude of importation. Left panels are estimates from the single-patch departmental model and right panels are estimated from the multi-patch departmental model. Each figure contains results from four departments, with the departments order from lowest to highest relative MASE as displayed in Figure 2.

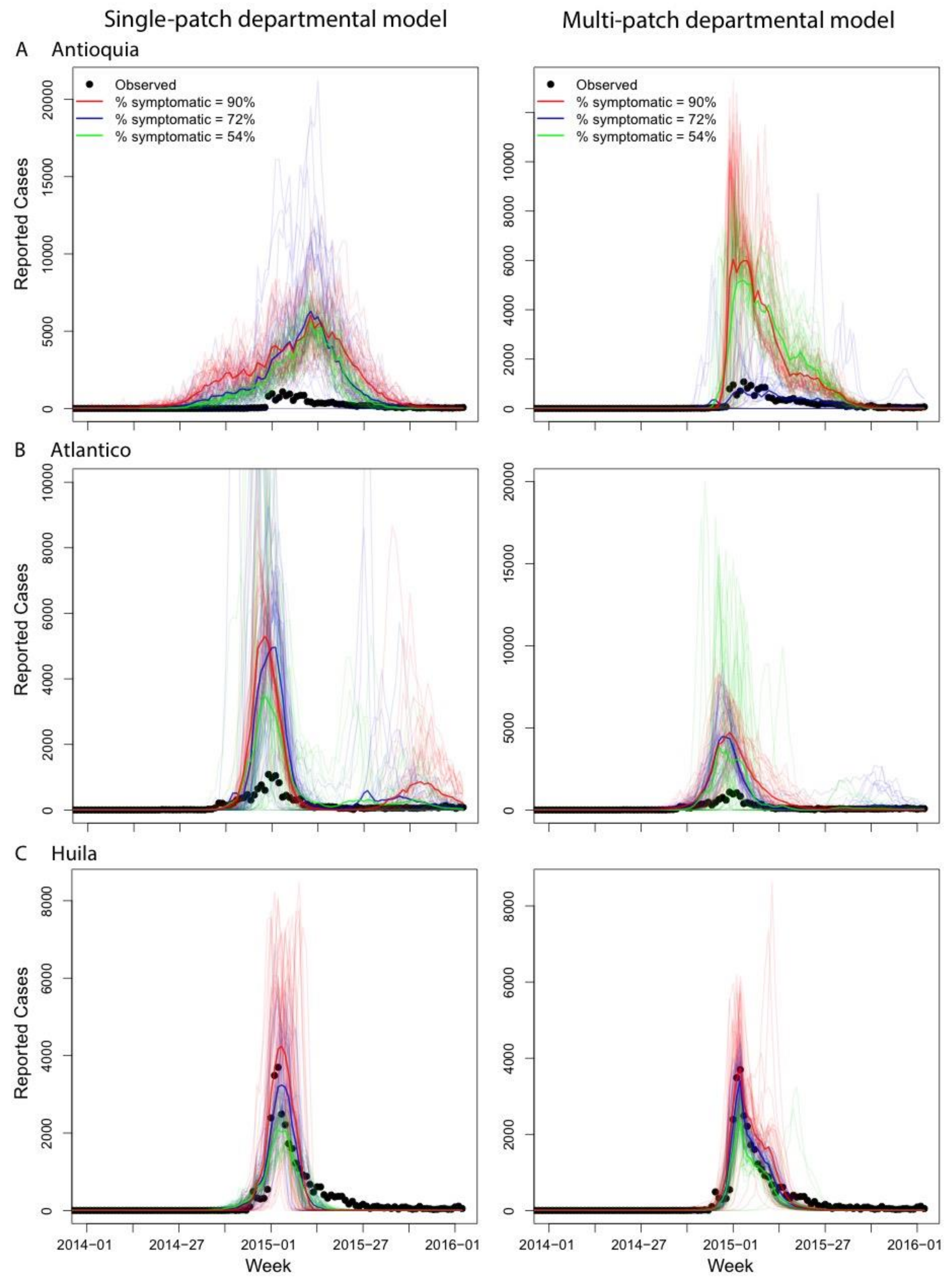

Figure S18. Comparisons of department-level results for single-patch and multi-patch models for three different symptomatic rates (0.54, 0.72, and 0.90). Black dots represent the observed time series, darker colored lines are the single best-fitting simulations, and lighter color lines are the other 40 top simulations.

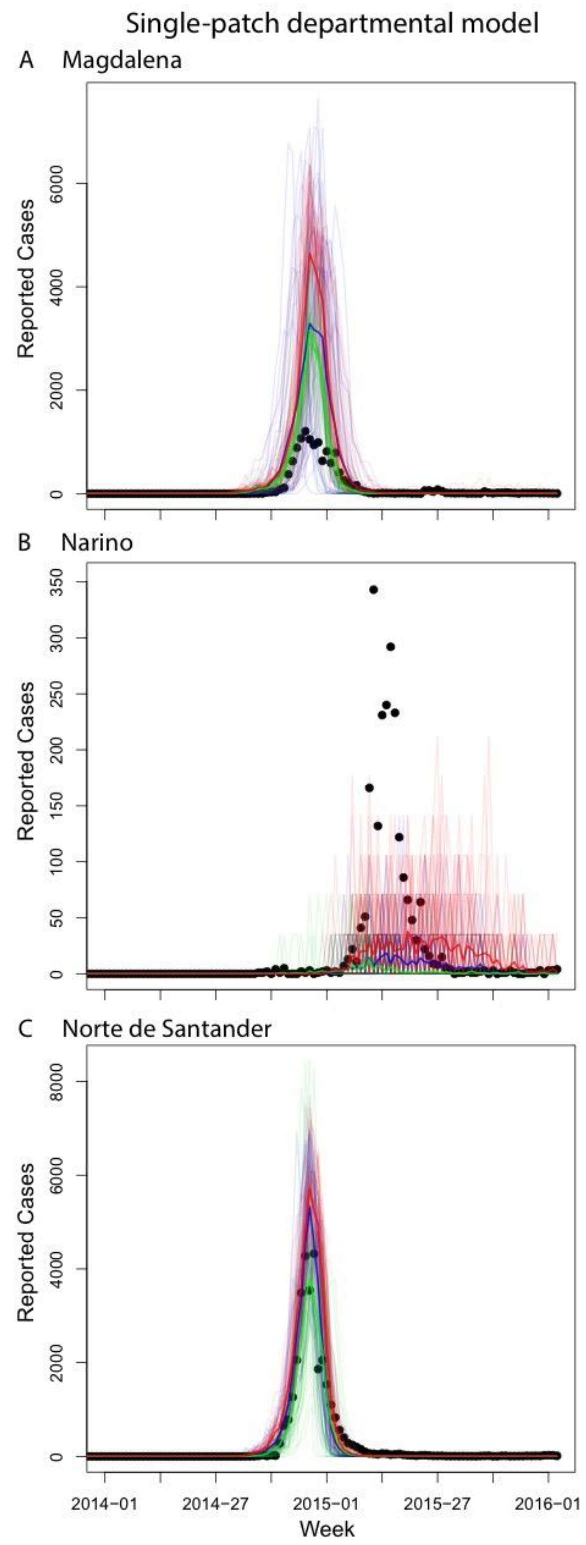

Figure S19. Comparisons of department-level results for single-patch and multi-patch models for three different symptomatic rates (0.54, 0.72, and 0.90). Black dots represent the observed time series, darker colored lines are the single best-fitting simulations, and lighter color lines are the other 40 top simulations.
